# Key Factors Impacting Performance Health During Growth and Maturation in Adolescent Competitive Aesthetic and Acrobatic Athletes: A Systematic Review

**DOI:** 10.1007/s40279-026-02416-5

**Published:** 2026-03-27

**Authors:** Alison S. Fitch, Jocelyn Mara, Felicity Lord, Gordon Waddington

**Affiliations:** 1 Research Institute for Sport and Exercise, University of Canberra, ACT 2617, PO Box 2032, Parap, NT 0804 Australia; 2 New South Wales Institute of Sport, 6 Figtree Drive, Sydney Olympic Park, NSW 2127 Australia; 3 Diving Australia, PO Box 23, Carina, QLD 4152 Australia; 4 Australian Institute of Sport, Bruce, Canberra, 2617 Australia

## Abstract

**Background:**

Aesthetic and acrobatic sports place high physical demands on youth athletes during critical periods of growth and maturation. These sports often involve intense training loads from an early age, which can influence physical development, injury risk, long-term health outcomes and performance.

**Objectives:**

The objectives were (1) to conduct a systematic review addressing key factors impacting performance health during growth and maturation in aesthetic and acrobatic high-performance youth athletes and (2) to investigate current methods for evaluating growth and maturation in this athletic population.

**Design:**

The Preferred Reporting Items for Systematic Reviews and Meta-analysis (PRISMA) guidelines were followed.

**Methods:**

The protocol was registered on the PROSPERO International prospective register (CRD42021261883). The electronic databases PubMed, Web of Science, SPORTDiscus and PsycINFO were searched for relevant studies up to 27 September 2025. Inclusion criteria were any sub-elite and elite athletes in aesthetic and acrobatic sports 18 years or under, a quantitative measurement of maturation and/or growth status/process in reference to the athlete’s health outcome. Two independent authors screened search results, performed data extraction and assessed risk of bias using the quality assessment tool developed by the National Heart, Lung, and Blood Institute (NHLBI) in collaboration with the Research Triangle Institute (RTI) international.

**Results:**

The results were reported on the basis of identifying 5 common themes amongst 65 studies and involving a total of 7782 participants; bone health; injury, illness and pain as outcomes; and anthropometric, biomechanical and training and performance as associated factors. Nutrition was also identified as a common interacting factor across themes. Growth and maturation measures and methods varied across studies. The review found that gymnastics training positively impacts bone health in prepubertal athletes, particularly in weight-bearing sites, although the risk of bone health injury increases around puberty possibly owing to later menarche, lower bone age and nutritional deficiencies. Dance studies show mixed results on bone health outcomes. Anthropometric and biomechanical factors including shorter stature, hypermobility and scoliosis and flexibility–strength imbalances may influence injury risk in association with being prepubescent. Later menarche, inadequate energy intake and discipline-specific bone development may contribute to tendon injuries, bone stress and overuse injuries in post-pubertal aesthetic and acrobatic athletes. Regular pre-pubertal gymnastics supports superior balance development. Training intensity can influence psychological stress and illness, particularly pre menarche, and male athletes respond differently to training than females during growth and maturation. Energy availability, along with psychological and behavioural factors may further affect injury, illness and performance outcomes.

**Conclusions:**

The factors contributing to performance health in aesthetic and acrobatic athletes during growth and maturation are inter-related and include anthropometric, biomechanical, training and nutrition. Earlier identification and appropriate timely prevention strategies are essential for performance health in aesthetic and acrobatic athletes.

**Supplementary Information:**

The online version contains supplementary material available at 10.1007/s40279-026-02416-5.

## Key Points

**Table:** 

Bone health in aesthetic and acrobatic athletes is supported pre puberty, but later maturation and low energy availability may increase the risk of bone stress injuries and overuse injuries post puberty.
Biomechanical factors, flexibility–strength imbalances and training intensity may influence injury and illness risk, further affected by psychological and behavioural factors such as anxiety and low awareness of relative energy deficiency in sport.
Continuous monitoring of growth and maturation is important for informing appropriate training programming and reducing injury and illness risk, given sex- and discipline-specific differences in response to training among aesthetic and acrobatic athletes.

## Introduction

Sports that require an entertaining component with artistic expression and athleticism are commonly known as aesthetic and acrobatic sports and include diving, gymnastics, dance, figure skating and synchronized swimming [1]. Aesthetic sports typically have set routines that require exceptional flexibility in the shoulders, hips and back, powerful jumps and repetitive tumbling or aerial rotations. Athletes must also show exceptional balance, coordination and good core control to enter the water safely, such as in diving, or land gracefully and in a technically sound landing onto the floor, apparatus or on ice [2, 3]. Competitions in aesthetic sports are judged and scored on the basis of the athlete’s execution of physical movements, whether or not the routine incorporates required elements (e.g. number of somersaults, twists, leaps, jumps, limb and dance positions, set time requirements), and the athlete’s physique, posture and presentation, as this can contribute to the ‘aesthetics’ of the performance [1, 4]. Training for athletes in aesthetic and acrobatic sports is highly repetitive in nature to enable mastery of performance skills at a high level. The intensity of training is both physically and mentally demanding; therefore, it is common for these athletes to specialize pre puberty to allow more time for building strength and refining their techniques for their performances [5–8].

Specialization, defined as ‘intense training in a single sport to the exclusion of all others’ [9] often begins in aesthetic and acrobatic sports by age 10 years, and can start as young as 6 years old [7, 8, 10, 11]. While early specialization is common amongst aesthetic and acrobatic athletes, it has been shown to lead to an added risk of injury, psychological burnout, illness and decreased longevity in their sport(s) [5, 7, 9, 12–14]. This can be attributed to the increased sport-specific long training hours, a greater rate of competition scheduling and less recovery time [7, 10, 11, 15]. As these young specializing athletes increase in chronological age (CA), they will also go through physical and physiological changes owing to the biological processes of puberty. This can vary widely between individuals during adolescence and the physical changes that occur to the body are defined as growth, whereas maturation is defined as the biological process towards the mature state [16–18]. Potentially this leads to a mismatch of differently growing body segments and tissues affecting neuromuscular control, which is important for aesthetic athletes owing to their complex aerial skills and balance requirements. This mismatch tends to occur predominantly during a growth spurt and significantly at peak height velocity (PHV) [19, 20]. PHV is the fastest upward growth in stature during adolescence and is also a time when bone mineral density decreases owing to delays in bone mineralization which can lead to a transient period of bone weakness and possible risk of bone stress injuries (BSI) [21, 22].

In aesthetic sports, the incidence of BSI is reportedly higher (10%) than in endurance (8%) and ball or technical sports (negligible) [12]. This may be attributed to the higher training and competition loads at a younger age among aesthetic sport athletes, compared with endurance and ball sport athletes, who tend to specialize later [11]. There is also a tendency for aesthetic athletes to mature later, reaching PHV at a higher CA, when training intensity and volume are much greater, which may further increase their susceptibility to growth-related injuries, such as BSI [23–25]. Furthermore, female athletes in aesthetic sports are more likely to have delayed menarche [26], which may predispose them to BSI owing to not reaching peak bone mass until a later CA [27]. In addition, factors such as distorted body image, relative energy deficiency in sport (REDs) and psychological stress have all been linked with aesthetic sport athletes [1, 28, 29]. Aesthetic sport athletes often have a low endomorphic body composition to enhance acrobatic performance and align with culturally specific physique standards used in judging and scoring [28, 30]. This has important consequences when peak bone mineral accrual occurs during these growing years and inadequate nutrition, body composition and endocrine function could negatively affect the potential for peak bone mass attainment, and they are also at risk of fatigue and underperforming. [10, 28, 30–32]. A recent study in academy soccer players found that suboptimal carbohydrate fuelling around periods of intense training was associated with increased bone resorption, potentially further compromising bone accrual during growth and maturation and increasing the risk of BSI [33].

Optimizing adolescent athletes’ full performance capacity and longevity in their chosen sport requires an understanding of the impact on health in their athletic journey, through growth and maturation [34, 35]. Performance health refers to the athlete’s physical, mental and social wellness that impacts their success in sport [36]. This understanding is required for best practice implementation of training and competition programs [10, 16].

The number of research reviews on youth sports health, mainly injury, through growth and maturation have increased in recent years to assist in understanding both the negative and positive effects on health through an athlete's prepubertal to post pubertal years [25, 31, 37–41]. There have been two reviews conducted including both aesthetic and acrobatic recreational and high-performance athletes in association with growth and maturation but concentrated to one type of health outcome, respectively: eating disorders [1] and overuse injuries [42]. To our knowledge, there are no systematic reviews that have explored only high-performance youth aesthetic and acrobatic athletes and their associated key health outcomes from early specialization as it relates to performance health [43].

Therefore, this systematic review aims to explore the factors impacting performance health during growth and maturation in competitive high-performance adolescent acrobatic and aesthetic sport athletes. A secondary aim of this review was to explore the current measures used to evaluate growth and maturation among this athletic population, to guide best practice for the selection and consistent application of appropriate assessment methods within high-performance acrobatic and aesthetic environments.

## Methods

The Preferred Reporting Items for Systematic Reviews and Meta-analyses (PRISMA) guidelines were followed to conduct this review [44].

### Information Sources and Search Strategy

The search strategy was developed and adapted from similar reviews on growth and maturation in adolescents [37, 38, 45]. The electronic databases PubMed, Web of Science, SPORTDiscus and PsycINFO were searched for relevant studies up to 23 July 2021. The search was then updated using the same four databases and search strategies, to include studies published between 23 July 2021 and 27 September 2025, ensuring the most recent evidence was incorporated (see online resource Supplementary File S2 for full search strategies).

The key search terms were selected on the basis of the objectives of the study and joined using the Boolean operator OR within each of the four themes:

- Sport: ‘diving’, ‘aquatic sports’, ‘Olympic sport’, ‘aesthetic sport’, ‘acrobatic sport’.
- Population: ‘adolescent’, ‘youth’.
- Health problem: ‘injury’, ‘illness’.
- Growth and maturation: ‘growth’, ‘maturation’, ‘peak height velocity’.

The function AND was used to join the themes. The following search terms were excluded using the function NOT: ‘scuba’, ‘deep-sea’, ‘recreation’, ‘obesity’, ‘diabetes’, ‘soccer’, ‘football’, ‘basketball’. Searches were limited to human participants (adolescent and children).

### Eligibility Criteria

Studies were included if the study:

- was written in the English language,
- was peer-reviewed,
- reported on data from competitive (sub-elite and elite) female and male aesthetic and acrobatic sport athletes that were ≤ 18 years of age*.* Sub-elite and elite athletes were defined as T1–E1, according to the Foundations, Talent, Elite, Mastery (FTEM) framework [46], or where participants were primarily involved in one sport at an institute, academy or dance school and represented their sport nationally or internationally at a junior or senior level,
- identified the participant’s chronological age,
- identified the sport within the results if comparisons were made (e.g. if aesthetic/acrobatic versus non aesthetic/acrobatic sports could be separated),
- reported a quantitative measurement of maturation and/or growth status/process in reference to the athlete’s health outcome. Measures of biological maturation could be represented by bone, sexual or somatic measurements and, as such, both actual and predicted assessments of maturity measurements may be included [47]. A measure of growth by change in stature by anthropometric data was required to show growth rate [38, 48],
- reported a health outcome in reference to their growth and/or maturation process; if a health problem (injury or illness) was reported, they could be stated as either:

(a) time loss, (b) medical attention, (c) all complaints as defined by Clarsen and Bahr, 2014 [49] or (d) conditions such as hypermobility or scoliosis (which are commonly associated with participation in aesthetic and acrobatic sports) [50] and involved a study design that was either prospective, retrospective, cross-sectional, case–control or case series observational.

Studies were excluded if the study:

- involved only non-aesthetic and non-acrobatic sports,
- investigated recreational or non-elite athletes,
- only reported on athletes over 18 years of age,
- did not report quantitative measures of maturation or growth in association with athlete health or
- was a systematic review, opinion piece, editorial or was not peer-reviewed.

### Data Collection and Selection Process

The reference management software, Endnote 20 (Clarivate, MA, USA http://www.endnote.com) was used to store relevant studies retrieved from database searches. These were then imported into the systematic review management system Covidence (Covidence Systematic Review Software, Veritas Health Innovation, Melbourne, Australia http://www.covidence.org) for screening.

Two authors (A.F. and F.L.) screened titles and abstracts independently for eligibility against the inclusion and exclusion criteria. A data extraction template was devised by A.F. in Covidence Software where the same two reviewers independently examined all full-text studies of eligible studies. If a disagreement occurred, a third independent reviewer (J.M.) was consulted to reach consensus.

### Data Items

The data extraction template included study type and duration and participant characteristics including sample size, chronological age, sex and sport. The type of control group used (sedentary, healthy match or different sport), their level of competition and/or their training history were also recorded. Growth and maturation measures and the methods of collection (e.g. stadiometer, scales, X-ray, age at menarche) used are presented in Table 1 and provided for all individual studies in online Supplementary File S1. Health outcomes were extracted and reported on in reference to either the control group, age reference standard, growth and/or maturation.

**Table 1:**
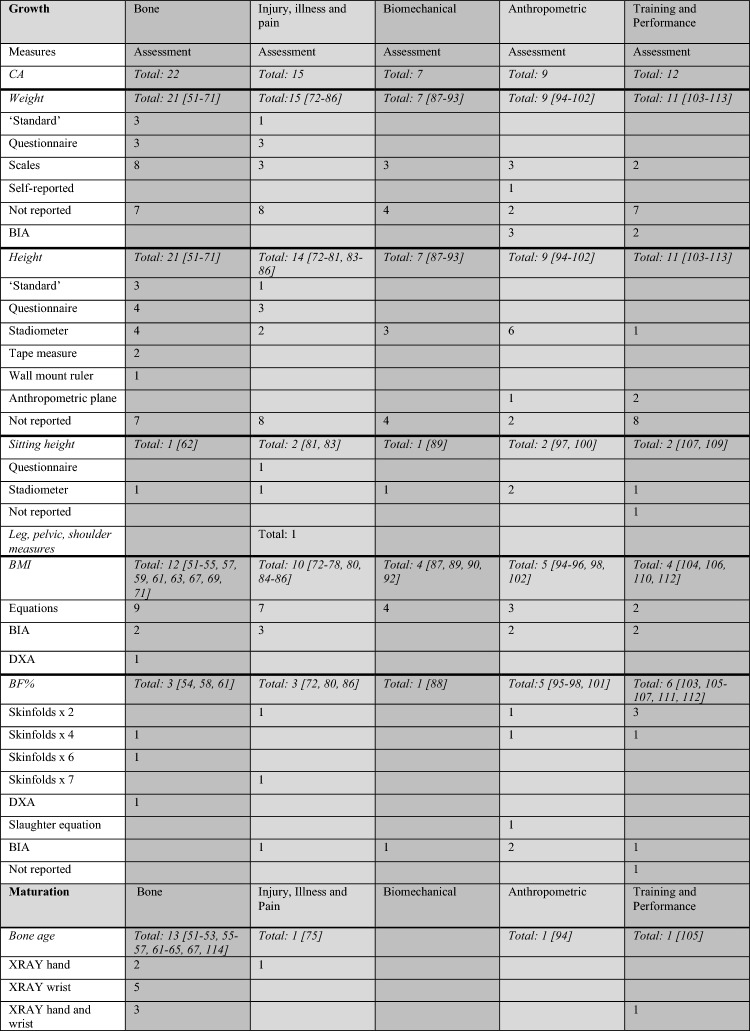

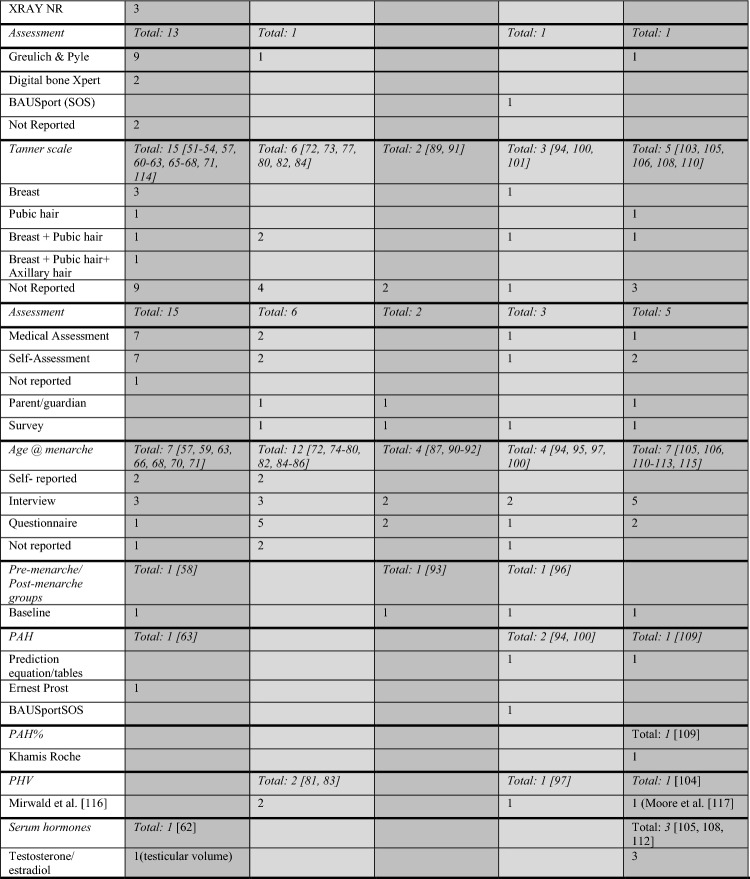
Growth and maturation measures in the studies included in this review

*BMI* Body Mass Index, *BIA* bioelectrical impedance analysis, *DXA* dual-energy X-ray absorptiometry, *BF%* body fat percentage, *PAH* predicted adult height, *%PAH* percent predicted adult height, *PHV* peak height velocity, *SOS* speed of sound

### Study Risk of Bias Assessment

Two Study Quality Assessment Tools created by the National Heart, Lung, and Blood Institute (NHLBI) and the Research Triangle Institute (RTI) International (2013) were used to evaluate potential limitations in study methods and implementation, including sources of bias, study power and confounding variables (https://www.nhlbi.nih.gov/health-topics/study-quality-assessment-tools). For observational cohort and cross-sectional studies, the criteria consisted of 14 questions; for quality assessment of case–control studies, the criteria consisted of 12 questions. Two independent reviewers (A.F. and F.L.) assessed each study, categorizing responses to each question as yes or no answers; not applicable (NA); not reported (NR); or cannot determine (CD). An overall quality rating of good, fair or poor was then given to each study on the basis of recommendations provided by the National Institutes of Health (NIH), Background: Development and Use of Study Quality Assessment Tools (https://internet-prod.nhlbi.nih.gov/node/80102). If a disagreement occurred, a third independent reviewer (J.M.) was consulted to reach consensus. The assessment process and final quality rating are presented in Table 1. The quality rating was not considered as an exclusion criteria; however, if a study was rated as ‘poor’, the evidence derived from that study was interpreted with less weighting than evidence derived from a study that was rated ‘good’.

### Synthesis of Results

A narrative synthesis of the results has been presented owing to the heterogeneous nature of study objectives, study designs and measures and methods of growth and maturation.

After review of the data extracted into Covidence, five main themes among studies were identified on the basis of commonalities among study titles, research aims as well as the measures collected. Two of these themes described common health outcomes described in literature and were categorized as: (1) bone health and (2) injury, illness and pain. The remaining three themes described common and key factors that influenced health outcomes (injury, illness) and were categorized as: (1) biomechanical factors, (2) anthropometrical factors and (3) training and performance factors. Nutrition, while not considered a ‘theme’ for the purpose of this review, was identified as a common interacting factor across themes.

## Results

### Study Selection

**Fig. 1:**
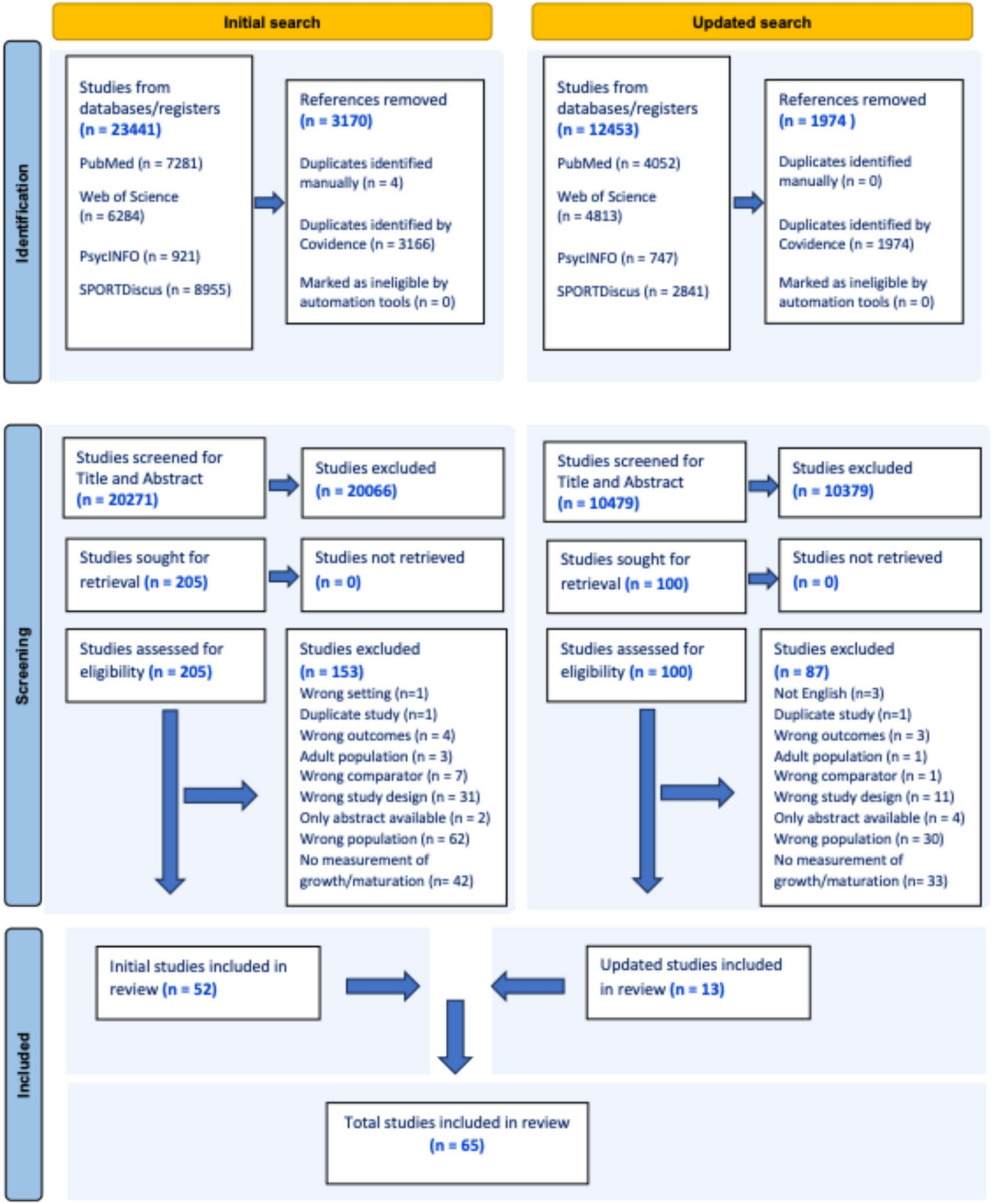
PRISMA flow chart

The initial search from four databases identified 23,441 potentially relevant articles. After removing duplicates, 20,271 articles remained for title and abstract screening. Subsequently, the full texts of 205 articles were assessed for eligibility, of which 52 studies met the inclusion criteria and were included in the initial review (Fig. 1). The updated search identified an additional 12,453 potentially relevant articles from the same four databases. After removing duplicates, 10,479 records remained for title and abstract screening. Of these, 100 full-text articles were assessed for eligibility, and 13 new studies met the inclusion criteria and were added to the original 52 studies. In total, 65 articles are included in this review (Fig. 1).

### Risk of Bias

The assessment process and final quality rating of the studies are presented in Table 2. There were no case–control studies (*n* = 13) that provided a justification of sample size. A total of 10 out of the 13 studies did not report if participants and controls were randomly selected for recruitment, and 8 out of 13 studies did not report if assessors were blinded to participants or controls. Three studies did not blind assessors for any variable, and another two studies only blinded for one variable (X-rays). Jaffré et al. [65] had six criteria not met (NO or NR) and Tournis et al. [61] met all criteria, with the exception of failing to provide a justification for sample size.

**Table 2:**
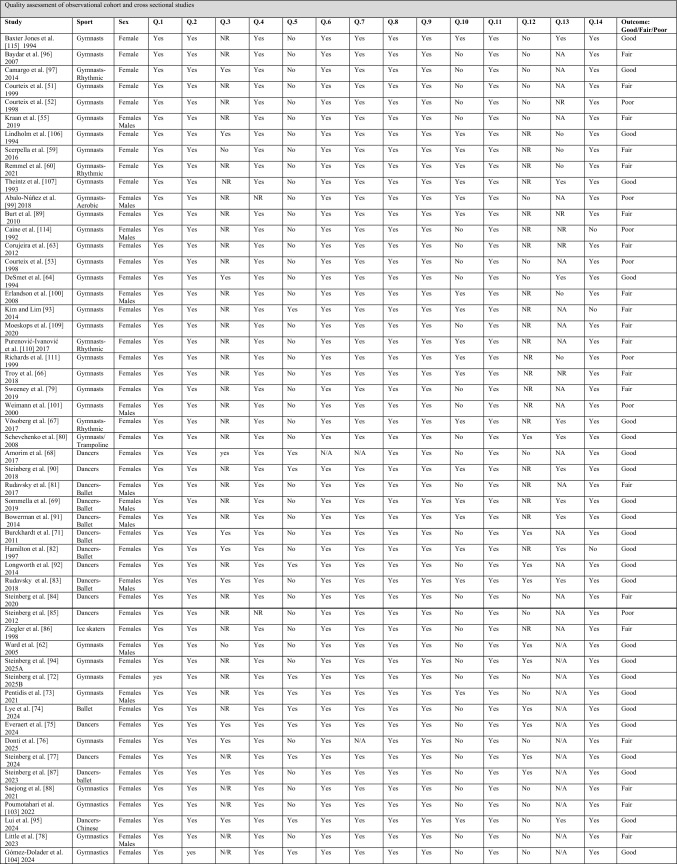

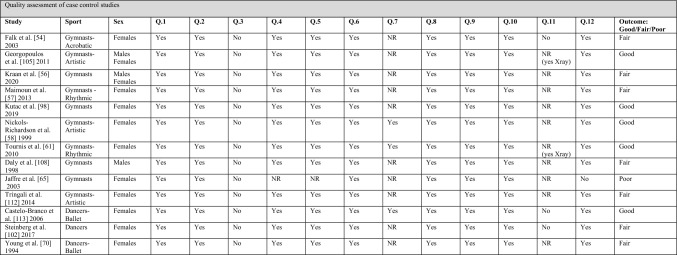
Risk of bias for the studies included in this review

Q1.Was the research question or objective in this paper clearly stated? Q2. Was the study population clearly specified and defined? Q3. Was the participation rate of eligible persons at least 50%? Q4. Were all the subjects selected or recruited from the same or similar populations (including the same time period)? Were the inclusion and exclusion criteria for being in the study prespecified and applied uniformly to all participants? Q5. Was a sample size justification, power description, or variance and effect estimates provided? Q6. For the analyses in this paper, were the exposure(s) of interest measured prior to the outcome(s) being measured? Q7. Was the timeframe sufficient so that one could reasonably expect to see an association between exposure and outcome if it existed? Q8. For the exposures that can vary in amount or level, did the study examine different levels of the exposure as related to the outcome (e.g. categories of exposure, or exposure measured as continuous variable)? Q9. Were the exposure measures (independent variables) clearly defined, valid, reliable, and implemented consistently across all study participants? 10. Was the exposure(s) assessed more than once over time? Q11. Were the outcome measures (dependent variables) clearly defined, valid, reliable, and implemented consistently across all study participants? Q12. Were the outcome assessors blinded to the exposure status of participants? Q13. Was loss to follow up after baseline 20% or less? 14. Were key potential confounding variables measured and adjusted statistically for their impact on the relationship between exposure(s) and outcomes(s)?

For observational and cross-sectional studies (*n* = 52), 39 studies did not report if 50% of eligible persons participated in their study. Sample size justification was only provided in 11 studies. Just over half of the studies (32 out of 52 studies) did not assess the outcome measures (‘exposures’) more than once. In total, 42 studies either did not blind the assessor or did not report on blinding. A total of 12 studies were under the recommended 20% for loss to follow-up after baseline, 5 studies were over the 20% and 5 were not reported. Only three studies did not adjust for confounding variables statistically.

### Study Characteristics

From the 65 included studies, characteristics were reported on the basis of the five themes: bone health; injury, illness and pain; anthropometric factors; biomechanical factors; and training and performance factors.

Collectively, 22 studies [51–71, 114] investigated the association of anthropometrics, nutritional practices and performance measures with bone health and reported on accrual of bone mineral density (BMD) and bone mineral content (BMC) and changes in bone geometry of common weight-bearing bones (radius, tibia, femoral neck and the lumbar spine). A total of 15 studies [72–86] investigated specific injuries, illness and pain, discussing tendon tissue changes that occur with and without pain, as well as scoliosis, and injuries to the lumbar spine and feet. These studies also examined age of menarche and menstrual health in relation to nutritional intake and low energy availability (LEA), as well as associations with eating disorders, anxiety about body weight and knowledge of REDs. Nine studies [94–102] concentrated on anthropometric measures and their association with nutrition and injury risk. Emphasis was placed on these athletes having lower body mass (BM), body mass index (BMI) and body fat percentage (BF%) with delayed bone age (BA) and maturation. Seven studies [87–93] focused on biomechanical parameters such as knee, hip and pelvic angles, hypermobility and scoliosis. These parameters were discussed in relation to injury risk and practice hours. A total of 12 studies [103–113, 115] examined the impact of training intensity and volume in association with body composition and athletic performance.

**Fig. 2:**
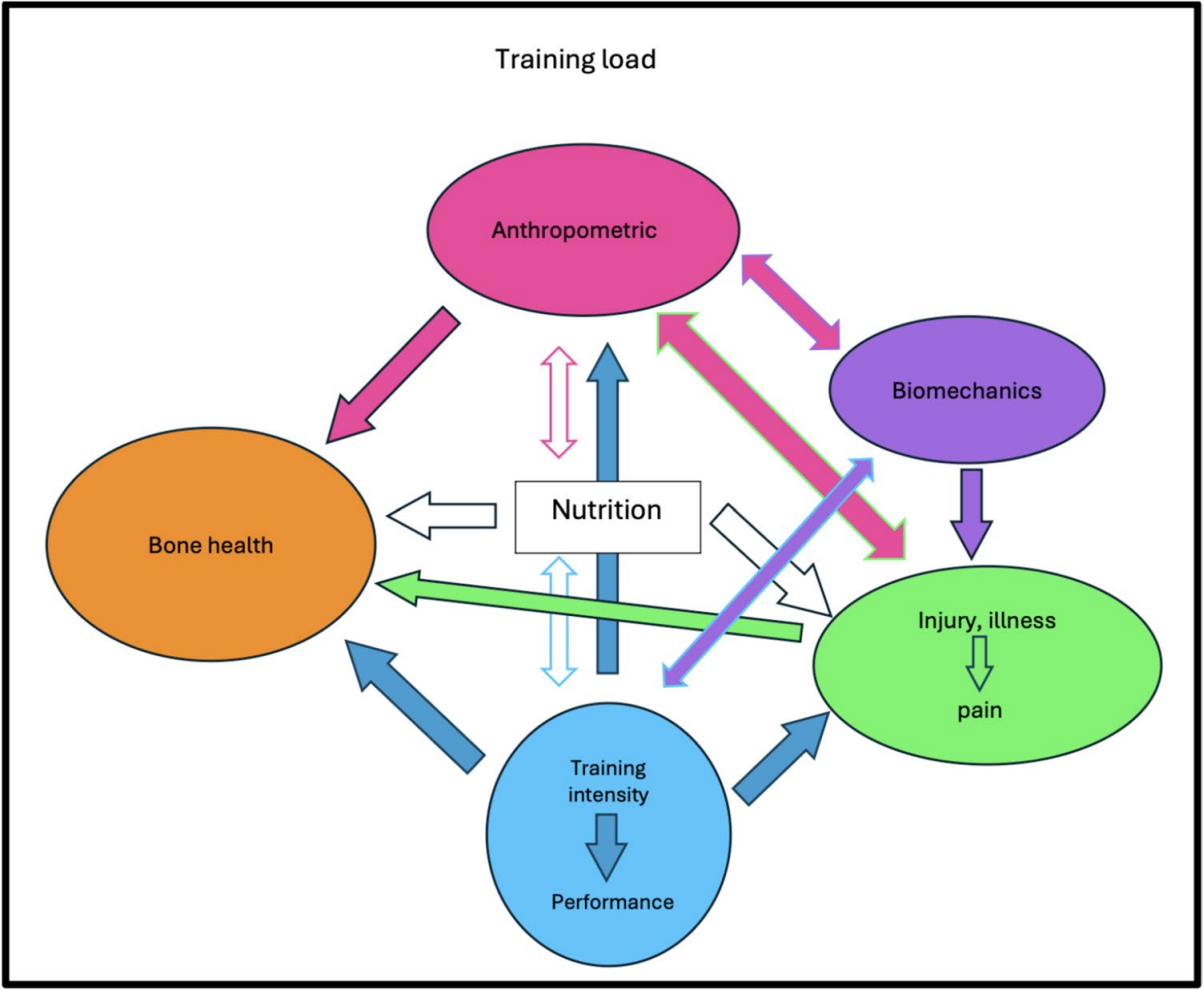
Interrelationships of studies’ key factors affecting health outcomes during growth and maturation whilst training for aesthetics and acrobatic sport. The five themes are related by the direction of the solid arrows. Each single arrow is pointing in the direction affecting the outcome. Double arrows indicate both outcome and/or factors interacting with one another. Nutrition was not one of the five themes, (non-solid arrows) but was a common factor throughout all themes, except biomechanical studies, interacting with other factors and the outcome. The width of the arrow estimates the approximate relative number of studies for each factor/outcome

The synthesis of results and discussion is also structured around these themes, and the relationships are shown in Fig. 2.

Studies predominately focused on gymnastic athletes (*n* = 45) and dance studies (*n* = 19). One study focussed on ice skating [86]. Most studies were conducted on female athletes (*n* = 50). A total of 14 studies included both male and female athletes, and one (*n* = 1) study was conducted on only male athletes [108]. Across studies, athletes trained on average 17 h per week in bone health, training and performance, and anthropometric studies (reported in *n* = 20, *n* = 11 and *n* = 8 studies, respectively), 14.2 h per week in biomechanical studies (reported in *n* = 4 studies) and 18.8 h per week in injury, illness and pain studies (reported in *n* = 13 studies). Details of these studies are provided in online Supplementary File S1.

Studies from all five identified themes (i.e. bone, 22 studies; anthropometric, 9 studies; biomechanical, 7 studies; training and performance, 12 studies; and injury, illness and pain, 15 studies) included common growth measures and chronological age (CA): weight, height, sitting height and BMI (Table 1). Biomechanical studies generally did not include BF%, with the exception of one study that measured BF% via a body composition analyser to assess specific physical characteristics related to scoliosis in elite rhythmic gymnasts [88]. Some studies did not report on how they measured anthropometric data (e.g. weight, height, sitting height) [51, 52, 54, 60, 65, 67, 69, 70, 73–75, 77, 78, 87, 88, 103, 104].

Bone health and training and performance studies were the two themes to include BA as measures of maturation. These were conducted via X-ray of the left hand and/or wrist using the Greulich and Pyle method: one study used the non-dominant hand and two studies used digital BoneXpert software. Only one other anthropometric study included BA as a measure of maturation using an ultrasound-based device (BAUSport) [94] (Table 1). Tanner Scale (31 studies) and age at menarche (34 studies) were the most used measures for maturation throughout all themes. Four studies used predicted adult height (PAH) [63, 94, 100, 109], four studies [81, 83, 97, 104] used PHV and only one study used percentage predicted adult height (%PAH) [109]. Serum hormone concentration levels (both estradiol and testosterone) were collected in four studies [62, 105, 108, 112] in which participants were categorized as pre or post menarche throughout the study. Some studies used up to four measures of maturation [63], whilst others only used one, most commonly age at menarche (Table 1).

## Discussion

To the best of our knowledge, this is the first review to investigate key factors affecting performance health during growth and maturation in aesthetic and acrobatic high performance youth athletes 18 years or younger. We identified five main themes from the 65 included studies: bone health (22 studies); injury, illness and pain (15 studies); biomechanical factors (7 studies); anthropometric factors (9 studies); and training and performance factors (12 studies). Two themes related to the main outcomes: bone health as well as injury, illness and pain. The other three themes were contributing key factors to and associations with these outcomes. These interrelationships are shown in Fig. 2.

Four sports met the eligibility criteria and were included in this review: artistic gymnastics (including acrobatic and aerobic), rhythmic gymnastics, dancing (including ballet, contemporary, modern, Chinese dance, dance sport and performing arts dancers) and ice skating. Research on other aesthetic sports, including diving, synchronized swimming and cheer and/or acrobatic sports did not meet the inclusion criteria on the basis of athlete age (often investigating older athletes aged 19–25 years) or they only reported on chronological age and did not investigate other growth and maturation measures in association with athlete’s health.

### Measures of Growth and Maturation

Growth and maturation measures and assessments varied across studies, making it difficult to aggregate and compare results. Considered the most reliable method for estimating maturity status in adolescence, BA provides valuable insight into a youth athlete’s readiness for training load by assessing the timing of growth plate closure and evaluating bone injury risk [10, 118, 119]. However, besides the 13 studies that investigated bone parameters specifically, only 3 other studies used BA as a measure of maturation [75, 94, 105]. This may be due to the ethical implications of exposing youth athletes to X-ray as well as logistical and resource difficulties, expertise required and the expense of conducting these over time. Perhaps an alternative in future research may be the use of predictive equations using anthropometric measures for establishing BA estimates [120].

Sexual maturation was measured using the Tanner scale across all study themes, and age at menarche was the most common measure used as a reference of sexual maturity for training and performance as well as the injury, illness and pain themes. It was common for studies to use two measures of sexual maturation, (e.g. Tanner scale and age at menarche) instead of another reference point for development, such as BA or %PAH. Growth patterns during adolescence are distinct with clear peaks and troughs and the upper and lower body growing at different rates [121]. This variability would suggest a combination of sexual and somatic methods to provide a more accurate representation of overall maturity stage. However, the appropriateness of assessing sexual maturity must be carefully considered, ensuring the measurements are conducted in a safe, secure and supportive environment that prioritizes protection and welfare of athletes [18]. In this review, only eight studies incorporated sitting height as a measure of growth, despite studies previously showing that sitting height is valuable to account for this differential in growth rates of the legs and trunk [116, 122].

There was a clear pattern in the choice of assessment methods based on either the outcome theme or the sport. For example, bone health studies frequently used BA and the Tanner scale, while studies on dance commonly used age at menarche and the Tanner scale. This is likely due to the nature of the variable they are studying and the uniqueness of the sport. Assessing BA is particularly relevant to bone health owing to understanding bone density and structural changes that may occur during each stage of puberty (Tanner scale), whereas dancing is particularly interested in age of menarche, as that is a pivotal stage of change in body composition, flexibility and possibly fatigue of the female athlete.

Inconsistencies arose within each measure, for example, the Tanner scale used three different measures: breast, pubic hair and armpit hair and sometimes a combination. The assessment was also different, varying from self-assessment, a medical professional conducting the examination or a parent. Other times it was not reported as to which scale was used for assessment. This makes comparison of results challenging, for example, an individual classified as stage 3 breast development may be alternately assessed as stage 4 pubic hair or vice versa. Again, the addition of a somatic measure of status and timing (%PAH and PHV) would assist in a clearer picture of overall development and comparison. Of note, the maturity offset method can systematically misestimate prediction of PHV, particularly in athletes who mature earlier or later than average. This limitation should be considered in the application for aesthetic athletes where growth, body composition and energy availability are closely linked to performance health and misclassification may affect training load prescription or injury risk management. Nevertheless, the method is non-invasive and practical in sport settings and when combined with consistent growth monitoring, it allows reliable relative comparisons of maturation within the cohort [123, 124].

### Bone Health

A total of 22 papers [51–71, 114] were included in the bone health theme and examined gymnasts and dancers through puberty revealing distinct patterns in BMD and BMC compared with controls.

Gymnastics training positively impacted bone health in youth athletes in regions such as the radius, femoral neck and lumbar spine [52, 57, 58, 60, 65, 67]. These studies demonstrated that gymnasts tend to have higher BMD and BMC compared with non-athletes, particularly in prepubertal stages and premenarchal years. This advantage tends to diminish if training is discontinued, noting that in the study by Scerpella et al. [59], many gymnasts reported injuries from gynaecological age 3 + years which led to ceasing training. So, although there are advantages to bone health in specific ‘loaded’ areas through training prepuberty, there also appears to be an injury risk when approaching puberty. This agrees with Patel et al. [34] regarding the increased risk of all injuries in both male and female elite gymnasts during the period of peak growth, when approaching 90% PAH [34].

In addition to higher BMD and BMC, some studies also showed a positive relationship in prepubertal and circa-menarche gymnasts with bone geometry: larger periosteal circumference, total bone area, cortical area, trabecular density and structural strength index (SSI) in the tibia and radius [61, 62, 66]. Cortical thickness and area were also positively associated with training years in prepubertal gymnasts [61]. In contrast, Kraan et al. [55, 56] found that symptomatic gymnasts’ wrists, with BAs between 12 and 14 years, had greater volume in the distal radial physis and thicker volar sections and more volar widening. Desmet et al. [64] also found stress-related changes in 10% of female gymnasts’ wrists with corresponding widening of the growth plate. Adaptation to bone loading from gymnastics training therefore may be favourable up until a point, but then increased bone density and structure may become more pathological with the more mature gymnasts facing a greater health risk. This could be due to years already in training, increases in hours spent training per week or the mismatch of growth of tissues during puberty. Weakening of soft tissue and a decrease in bone remodelling may further contribute [25, 31].

Lean tissue mass was a significant predictor of BMC and BMD in female pre-pubertal gymnasts in the lumbar spine, femoral neck and radius [53, 67]. Muscle cross-sectional area was also increased in both pubertal male [62] and female gymnasts (transition zone) [61] with associated higher jump performances [60, 67]. In addition, Sommella et al. [69] found that in female and male ballet dancers with over 3 years of training and an average of 23 h/week training per year, BMD of the legs improved significantly with a positive correlation with strength in the ankle plantar flexors. In contrast, lower or similar BMD and BMC were seen in pre- and post-pubertal dancers at the forearm, femoral neck and lumbar spine compared with age- and sex-matched controls, and dancers had similar bone density to anorexic girls at non-weight bearing (NWB) sites [68, 70, 71]. This agrees with research by Munoz et al. [125] suggesting site-specific loading in rhythmic or dance athletes, as they may not have enough mechanical loading for additional bone mass accrual in the upper or lower extremities (as compared with gymnasts). There may also be an associated negative energy balance from poor diet [31].

Gymnasts and dancers in these studies were shorter, lighter and had a lower BMI compared with controls, along with reduced fat mass and BF% [51, 52, 54, 57–63, 67, 68]. In addition, some studies reported delayed Tanner stage and age at menarche in these athletes compared with controls [57, 67, 68, 71], whilst others noted a lower mean BA [51, 53, 126]. As a result, there appears to be a trade-off between achieving optimal bone loading before puberty and an elevated risk of injury, possibly owing to delayed BA and menarche, which could stem from the pressure to maintain a lower weight. These delays may be linked to reduced energy availability by the increased intensity of training and inadequate nutrient intake for bone remodelling (calcium), leading to decreased leptin levels [51, 52, 54, 58, 60]. Males appear more immune to this injury risk owing to greater muscle gains for protective advantages [27, 53, 62], although limited studies were available. Of note, the smaller size, delayed age at menarche and BA observed in aesthetic athletes may also reflect selection bias towards later-maturing individuals. Some studies have indicated that these traits are present regardless of training volume [127–130].

### Injury, Illness and Pain

The injury, illness and pain theme included 15 studies [72–86] exploring the impact of injury, illness or pain through growth and maturation. Six studies focused on gymnastics [72, 73, 76, 78–80], eight on dance [74, 75, 77, 81–85] and one on ice skating [86]. Tendon health was investigated in four studies on dancers [77, 81, 83, 84] and two studies on gymnasts [72, 73]. Rudavsky et al. [81, 83] reported no significant tendon pathology among growth phases but noted increased patellar tendon thickness and disorganized echoes in post-PHV dancers. Steinberg et al. [84] found a higher prevalence of certain echo types in post-menarche dancers' in Achilles tendons while noting no pain difference between pre- and post-menarche groups. Although no injury was reported in these studies, the findings highlight the potential vulnerability of the tendons and longer-term injury risk post puberty with these tissue structural changes. Tendon overuse injuries, particularly in ballet, have been well documented. These injuries often result from repetitive jumping and landing movements which cause microtrauma in tendon tissues owing to high impact and are seen post puberty [37, 38, 131]. Recent evidence from Steinberg et al. [77] found patellofemoral pain (PFP) in 49% of the dancers in their study. Further, post-pubertal dancers with PFP had less muscle strength, low radial and tibial bone properties, poorer tendon structure, greater en pointe and hip external rotation (ER), range of motion (ROM) and pain with jumping and weight-bearing on a flexed knee. In comparison, among gymnasts, Pentidis et al. [73] found that imbalanced muscle–tendon adaptation pre puberty and reduced bone density are associated with heightened susceptibility to PFP, tendon strain and overuse injuries. In addition, Steinberg et al. [72] found no difference with maturation stage or pre or post menarche but a higher prevalence of PFP amongst artistic gymnasts compared with acrobatic gymnasts, which was associated with greater weekly training hours per week, reduced hip strength and greater ankle flexibility.

Sweeney et al. [79] found low back pain at post menarche was not affected by hip flexibility or activity level in adolescent gymnasts, but Steinberg et al. [85] reported less hip ER ROM in post-menarche dancers with back pain. In addition, hyper-abduction hip ROM and dance activity level were seen as predictors of ankle and foot tendinopathies post menarche. Scoliosis, plantar flexion (PF) of the ankle and dance techniques were variables associated with back injuries [85]. Hamilton et al. [82] conducted a 4-year longitudinal study on dancers (Tanner stage 3–4) and found those with minor injuries had more anatomical problems and dynamic deficits than those who were uninjured. Over the course of the 4-year study, 83% sustained injuries in ballet class and 64% developed additional injuries as the study progressed. These findings suggest that hip ROM is only one component of injury risk and other factors such as training intensity, biomechanics/technique, muscle fatigue, lower skeletal age and hours of practice can all contribute to greater injury risk post menarche [75]. Scoliosis seems to stand alone and be a greater risk factor for injuries in the younger years (< 12 years) [85], possibly due to a combination of less dance experience and greater loads on specific muscles and joints in a more immature body. This highlights the importance of identifying scoliosis early in dancers and implementing proactive management strategies to minimize injury risk and support long-term physical health.

Three studies examined injury risk associated with delayed menarche and puberty in gymnasts and dancers [80, 82, 84]. Dancers who experienced average or late menarche had a higher injury rate (45%) compared with those with early menarche (38%), with 10% of the former group developing stress fractures [85]. The low estrogenic state associated with this delay [80, 82] could lower bone density and muscle recovery, making these athletes more susceptible to injury, especially fractures. Furthermore, studies assessing nutritional practices [82, 86] highlighted the inadequate energy intakes among dancers, gymnasts and ice skaters, particularly in terms of calcium and iron intake. This under-fuelling further emphasizes the risk of bone injuries, as LEA can both limit the essential nutrients required for adequate bone repair and elevate bone resorption during periods of intense training, contributing to lower BMD and greater susceptibility to overuse injuries [33]. Supporting this, studies in both gymnastics and dance have reported that over half of the athletes in the studies show markers of LEA such as delayed BA, menstrual irregularities or amenorrhea and lower BMI and they are at increased risk of developing LEA with increasing age [74, 75].

Other common anthropometric characteristics observed in these aesthetic athletes included lower height, weight, BF% and, again, BMI compared with control groups [80, 82, 84]. Higher rates of bulimia were reported in dancers with overuse injuries. Those with clinical eating disorders (ED) showed anatomical asymmetries of the leg: length, flexibility and turnout of the knee. These physical imbalances were associated with a higher incidence of injuries leading to more missed classes [82]. Similarly, dancers and gymnasts had a higher likelihood of developing an ED than their less-trained peers [76]. Little et al. [78] further reported increased body mass concerns, anxiety (assessed via Generalized Anxiety Disorder 7-item [GAD-7] questionnaire) and body dissatisfaction amongst gymnasts, while Donti [76] reported that 80–90% of artistic gymnasts were unaware of LEA or REDs concepts, highlighting limited awareness of health risks associated with under-fuelling. Furthermore, illness was identified in one study, with gymnasts having a higher infection rate [80], which may reflect the additional stress of training on an under-fuelled growing body, making the immune system more compromised and further delaying recovery.

Of note is the influence of familial genetics and the predisposition of these athletes to be smaller, lighter and delayed in menarche like their mothers [80]. Zeigler et al. [86] found no relationship between menstrual state and percentage body fat in ice skaters, and Steinberg et al. [85] found no association between BMI and injuries in stages of maturation in dancers. This may suggest that overall body composition within each aesthetic sport and different nutritional requirements can influence injury risk.

### Biomechanical Factors and Injury Risk

In total, seven studies assessed biomechanical factors in sport and related these to injury risk [87, 88, 91–93] and level of skill [89, 90]. Post-menarche dancers faced higher risks of non-contact anterior cruciate ligament (ACL) injuries owing to increased knee loads during single-leg drop landings, with contributing factors including decreased maximum knee flexion and increased knee abduction angles [93]. Specific knee and pelvis angles were linked to substantial injury risk in 16-year-old ballet dancers, while maturation, age, height and body mass had minimal influence [91]. This suggests that technique and biomechanics are contributors to injury risk, with increased oestrogen post menarche linked to greater knee laxity [132, 133]. Focusing on neuromuscular control and hip-strengthening interventions may assist in reducing this risk for ACL injuries [133, 134].

Steinberg et al. [87] examined maturation, joint ROM and muscle strength in young female dancers (12–14 years) and found that greater joint ROM was negatively correlated with muscle strength, particularly between en pointe ROM and ankle PF and DF as well as between hip ER and knee extensors. Pre-menarche dancers demonstrated greater joint ROM but lower muscle strength than post-menarche dancers, suggesting that excessive flexibility without corresponding strength development may compromise joint stability and proprioception, thereby increasing susceptibility to overuse injuries [135–137]. Interventions therefore need to target muscle strength and control around hypermobile joints with neuromuscular screening and careful management of training loads across stages of maturation [45, 87, 136].

Hypermobility (Beighton’s score > 4) was found in 21/30 adolescent ballet dancers with a statistically significant higher prevalence of scoliosis in dancers (30%) compared with non-dancers (3.33%) and more hours of dancing unsupervised contributing to increased scoliosis [92]. Saejong et al. [88] found rhythmic gymnasts increasingly susceptible to scoliosis with years of intensive training, and higher age, height, weight and BF%. In addition, asymmetrical lateral flexor strength was more severe in those with scoliosis. Gymnastic rotation skills and wrist impacts had greater variability in pre-pubertal gymnasts with a higher level of participation [89], indicating that higher training volumes without proper technique, especially with asymmetrical positions (common in ballet) and being skeletally immature, can put the athlete at risk of musculoskeletal stress and adverse structural changes (i.e. further progression of scoliosis).

Ground reaction forces were found to be higher in international-level gymnasts than national gymnasts [89], which may reflect training intensity and more complex routines but does support the possible need for inclusion of adequate recovery and rest, as mentioned previously, to meet these extra demands on the bodies, especially if not yet skeletally mature.

In one study, hip ER and ankle foot en pointe were significantly improved with 1 year of dance training [90] in pre- and post-menarche groups (from year 7 to year 8). The non-menarche group had greater en pointe ROM than the post-menarche group after 1 year. Thus, the relationship between hours of practice and adaptability can be positive but needs to be supervised and progressed slowly to control for these extreme ranges, avoiding negative health consequences as maturity progresses [138].

### Anthropometric Factors and Injury Risk

Collectively, nine papers investigated anthropometric measures through growth and maturation and injury risk [94–102], with nutrition as a common interacting factor for this risk. Dancers’ structural characteristics such as scoliosis, hind-foot varum, less ankle PF, greater ankle dorsiflexion (DF) and more knee joint flexion increased injury risk for patellofemoral pain syndrome (PFPS) regardless of the age of dancers [102]. There was also a trend for prevalence at the knee joint of a hypermobile patella [136].

An excessive *Q* angle in aerobic gymnasts, especially the left *Q* angle, showed a link to higher odds of lower-limb injuries with its effect varying according to the athlete’s weight [99].

In addition, imbalanced weight distribution between left and right legs can influence injury along with training longer than 2 h/day and having a previous injury [99]. These two studies emphasize that with a combination of asymmetrical loading patterns, intensity of training and less control of joint ROM (which all increase through maturation) can influence an athlete’s chance of musculoskeletal injury, particularly PFPS [139, 140].

Along with the above factors for injury risk were the common anthropometric factors of lower body mass, BMI, BF% and delayed BA in female athletes [96–98, 100]. Gymnasts tended to have shorter adult height, with a later age of menarche and delayed Tanner stage 5 than other sports but normal adrenal maturation [100, 101]. Steinberg et al. [94] reported that artistic gymnasts were shorter than rhythmic and acrobatic gymnasts despite similar BMI and body fat and also demonstrated lower tibial bone strength, which combined with high impact loading may increase susceptibility to BSI. Among adolescent gymnasts (> 13 years), BA was lower than their CA and final height predictions were greater for rhythmic gymnasts than for artistic gymnasts, identifying the possible discipline-specific effects on growth. In adolescent dancers, the average age of menarche was 12.5 years, with 22.1% reporting secondary amenorrhea and having lower BF% than those with normal menstrual cycles [95].

As discussed previously, these factors can all influence increased risk of injury and illness owing to lower BMD and less-than-optimal recovery, possibly due to inadequate fuelling to maintain smaller body mass and BF% [141, 142]. Interestingly, adrenal maturation was seen as normal, possibly reflecting the influence of normal protein consumption in athletes seen in two studies, [96, 101] which has been linked to higher levels of dehydroepiandrosterone sulphate (DHES) in adrenal maturation, [143] but athletes were more likely to have lower vitamin and mineral intake [96, 101]. Calcium and vitamin D are both critical in bone density development during puberty to reach peak bone mass, and this deficit could then explain the delays in BA and consequential fracture risk as previously mentioned [144].

Of importance, and in comparison with females, male gymnasts’ BA corresponded to their CA, a normal progression of puberty, and a greater increase in fat free mass (FFM) during puberty than females [101]. This highlights sex differences and the natural development of FFM (i.e. lean tissue mass) in males during puberty, which is possibly protective against bone and joint injuries and counteracts the lower BF% seen in both male and female aesthetic athletes [145, 146]. More research on male aesthetic athletes is needed as it relates to body composition, nutritional intake and injury risk.

### Training and Performance Factors and Illness

Twelve studies examined the impact of training intensity in association with body composition and athletic performance among elite gymnasts [103–111, 115] and one ballet cohort [113]. Participants in these studies trained at national gymnastics or dance centres, averaging 17 h/week, while control groups included recreational athletes or peers from public schools.

Cortisol levels were analysed, revealing that female gymnasts had higher morning cortisol levels and greater psychological stress compared with controls, while males experienced increased stress but without elevated cortisol levels [105, 108]. Given earlier specialization and the heightened pressures from training and competition at a young age, monitoring mental health and stress regulation is important in these athletes [147]. Chronically high cortisol levels can lead to immune suppression and impaired recovery, which can compromise long-term health and performance, making effective management essential [148].

Pro-inflammatory markers were elevated in female gymnasts; however, these markers were potentially mitigated by higher circulating estradiol levels post menarche [112]. However, as it is common for these athletes to have delayed menarche, the period before estradiol levels rise is extended, potentially prolonging the pre-menarche risk phase.

Greater force development was shown in more mature gymnasts compared with their less-mature or lower-level trained peers [103, 104, 109–111]. This enhanced performance was associated with both higher training volumes and increased strength with maturity, despite their smaller body mass relative to peers with lower training volume [111]. Regular gymnastics training pre puberty appears to maintain elevated neuromuscular performance more so than in untrained females [103], and strength and balance improve significantly after PHV with maximal isometric strength and countermovement jump as key predictors of static balance [104]. Gymnasts, as previously discussed in other themes, were lighter and shorter than other athletes, with delayed BA and slower growth rates [105–107, 115]. Height or body mass were indirectly associated with centre of pressure (CoP) excursion [104], suggesting that other variables such as strength or maturation may influence this postural stability, as noted previously.

Gymnasts experienced a later average onset of menarche (14.2–14.5 years) compared with control groups, [106, 107, 115] while dancers had a slightly earlier onset (12.4 years) but displayed more irregular menstruation [113]. Maternal age of menarche was seen to influence menarcheal age of the athletes here as well, aligning with previous observations seen in the injury, illness and pain theme [115]. The combination of training intensity, nutrition, sport requirements and genetics most likely plays a role in the development of these physical attributes [149].

### Summary of Findings

The key findings across bone health, injury and illness risk; anthropometric characteristics; biomechanical factors; and training responses in aesthetic and acrobatic youth athletes are presented in Table 3.

**Table 3:**
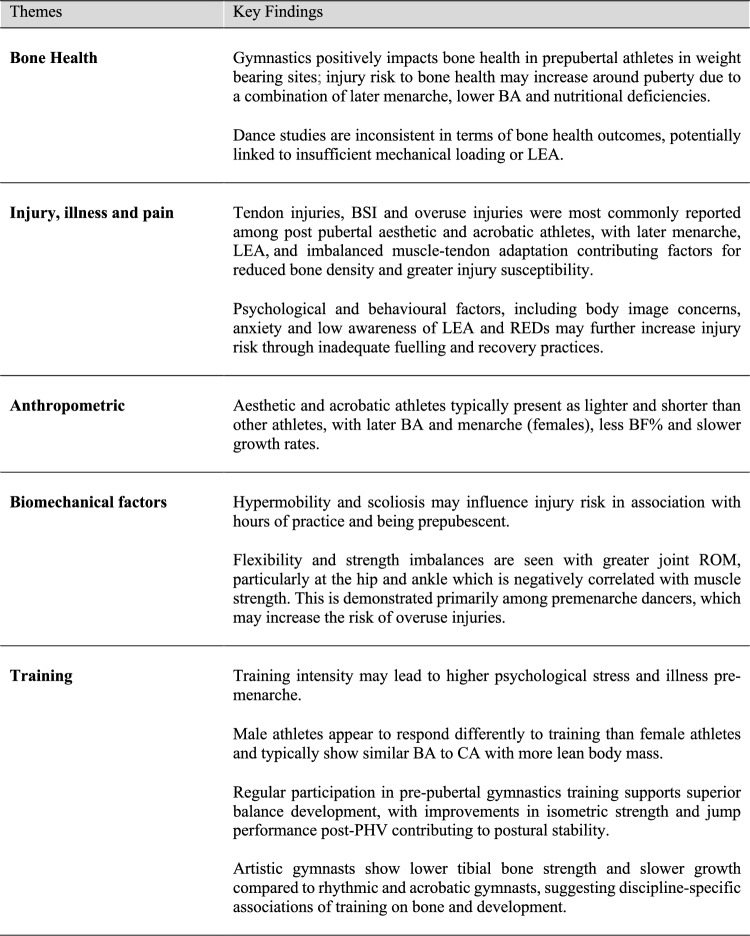
Summary table

*BA* bone age, *LEA* low energy availability, *BSI* bone stress injury, *REDs* relative energy deficiency in sport, *BF%* body fat percentage, *ROM* range of motion, *CA* chronological age, *PHV* peak height velocity

### Implications for Performance Health Practice

Health performance staff should adopt a multimethod approach to monitor growth and maturation in youth aesthetic and acrobatic athletes. Best practice should include assessing BA, menarche status and somatic growth measures (height, weight, sitting height) to estimate maturity status and timing (%PAH and PHV), and track growth velocities for height and weight. Any assessment of sexual maturity must be conducted in a safe and supportive environment that prioritizes athlete welfare, and sensitivity should be used in any growth and maturation measures given the high prevalence of REDs within aesthetic sports. Guardian consent is required for youth athletes under 18 years old, along with agreement from the athlete’s performance support team [150]. Consistent longitudinal monitoring of growth, integrating maturity indicators to enhance accuracy, will support training loads that are appropriately matched to each athlete’s biological stage and help identify periods of elevated growth-related risk for injury or illness. Importantly, members of the athlete’s performance support team (including doctors, physiotherapists, coaches and dietitians) should have access to appropriate resources and receive educational support to understand the variations and accuracy of the methods used. This knowledge is essential for the effective implementation of athlete management strategies to prioritize health. Providing feedback to parents/guardian and athletes can further support these strategies and optimize performance health [18].

In addition to tracking overall training volume or hours, monitoring training intensity by the specific type of exercise or technique performed is recommended. This can help identify injury risks more effectively. Particular consideration should be given to reducing training load during early post-menarche years in female athletes. The addition of neuromuscular intervention programs would also be beneficial, as biomechanical changes during puberty can contribute to an increased risk of injury in these athletes.

Early identification of conditions such as hypermobility and scoliosis in pre-pubescent athletes, followed by ongoing monitoring through growth and maturation, is strongly advised. Timely detection through targeted spinal screenings and management of these conditions with targeted postural conditioning could reduce the likelihood of structural changes that arise post puberty and are associated with subsequent injuries and missed training.

Given the potential for delayed BA, later menarche, menstrual irregularities, increased risk of BSI and disordered eating in this population, together reflecting the broader spectrum of REDs, it is essential to closely monitor energy intake, ensure adequate consumption of vitamins and minerals pre puberty and track menstrual cycles. Targeted nutritional support and education can optimize both short- and long-term bone health, mitigate LEA and reduce the likelihood of developing EDs. These prevention strategies can also help preserve musculoskeletal and psychological health, promoting safe long-term participation in aesthetic sports.

Lastly, ongoing monitoring of stress and recovery across maturation is important, particularly as training intensity and competition demands increase. Ensuring adequate recovery can help protect both physical and psychological health during vulnerable stages of development and contribute to sustained participation, performance and wellbeing in aesthetic and acrobatic sports.

### Limitations

Variability in study designs, athlete samples chosen and participant numbers as well as inconsistent methodologies for growth and maturation measures make it difficult to compare and contrast studies. For example, pre and post menarche is not the same as pre and post puberty.

Studies predominantly examined female participants, which means that results cannot be generalized to male athletes, and training loads and intensities were wide and varied across studies. There were also differences in study durations and time in season of data collection, which could all bias associations.

## Conclusions

The aim of this review was to identify which factors influenced performance health during growth and maturation in aesthetic and acrobatic athletes. Aesthetic and acrobatic athletes tend to have lighter body mass and delayed bone age and menarche compared with other athletes. Puberty increases the risk of bone stress injuries owing to delayed maturation and potential nutritional deficiencies. Bone health is supported in gymnasts pre puberty, but these athletes face an increased risk of bone, tendon and overuse injuries post puberty. Flexibility and strength imbalances, particularly of the hip and ankle, and discipline-specific effects of bone development may further increase injury risk. Regular pre-pubertal gymnastics training supports superior balance development, with post-PHV improvements in strength and jump performance contributing to postural stability. Anthropometric and biomechanical factors, alongside energy availability and psychological and behavioural factors may also influence injury and illness susceptibility. Ongoing research should focus on monitoring more male athlete aesthetic cohorts, consistent longitudinal growth and maturation measures, energy intake and stress/recovery levels from pre-puberty for deeper insights into performance health. Understanding performance health during growth and maturation can assist the development of training programs for longevity of career and the progression to elite-level sport in aesthetic and acrobatic sport athletes.

## Supplementary Information

Below is the link to the electronic supplementary material.

Supplementary file1 (PDF 399 KB)

Supplementary file2 (PDF 100 KB)

## Declarations

### Availability of Data

The authors confirm that the data supporting the results of this review are available within the article and its supplementary materials.
