# Supplementary material for: Key Factors Impacting Performance Health During Growth and Maturation in Adolescent Competitive Aesthetic and Acrobatic Athletes: A Systematic Review
Source: Sports Med. 2026 Mar 27;56(7):1701–21. doi: 10.1007/s40279-026-02416-5 (PMC13388506; doi:10.1007/s40279-026-02416-5)
Supplement: Supplementary file 1 — Supplementary file1 (PDF 399 KB) [file 40279_2026_2416_MOESM1_ESM.pdf]

Alison S Fitch, Jocelyn Mara, Felicity Lord, Gordon Waddington. Corresponding author: Alison Fitch Email: sportsphysio@alifitch.com ali.fitch@canberra.edu.au Research Institute for Sport and Exercise, University of Canberra ACT 2617, Australia

Supplementary file S1. Summary of Individual Studies

First Author	Sport/ Sex	Type/Duration	Number	Age	Training	Measures<br>Growth	Maturation	Methods<br>Assessment	Assessment	Results
Bone										
Courteix et al. [51] 1999	Gymnasts<br>Female	Cross-sectional comparative	N=60:<br>32 gym<br>12 swim<br>16 controls	Mean years:<br>10.15 gym<br>10.6 swim<br>10.5 control	Gym: 3 yr training history,<br>10-15hrs/week, 5 day/week plus comps	CA, weight, height, BMI	Bone age, Tanner scale	Calcium intake, Frequency of food questionnaire, Training load	BMD, BMC, FM, FFM (DXA) whole body measured; during training season.	*FM was sign lower in gym c/w other groups, wt higher in swimmers c/w gym<br>*No sign diff in Ca intake, below recommended<br>*Mean BMD sign higher in gym in R, FN, WT. BMC sign higher in gym for UDR (p<.01) and wt (p<.01)<br>*Head BMC and BMD correlated sign (p ~.001) with BA, ht, wt, LM, FM & WB BMC.<br>*Slopes OR for head/whole body BMC or BMD were sign lower in gym (p <.05)
Courteix et al. [52] 1998 (A)	Gymnasts<br>Female	Cross-sectional	N= 41<br>18 gym<br>10 swim<br>13 controls	Mean years:<br>10.4 gym<br>10.5 swim<br>10.7 controls	Gym: 3 yr training history,<br>10-15hrs/week, 5 day/week plus comps	CA, weight, height, BMI	Bone age, Tanner scale, prepubertal	Calcium intake, Frequency of food questionnaire, Training load	BMD, BMC,FM, FFM, BMI (DXA); during training season	*Lower mean BA in gym vs other groups and were smaller, slender and leaner with smaller % BF<br>*No sign diff in Ca intake<br>*Mean BMD for gym were sign higher (not WB BMD, trochanter BMD), values at R, trochanter and FN were above normative values<br>*Slope OR of wt and WB BMD was sign higher in gym<br>*BMC values sign higher in gym for all sites, except trochanter
Falk et al. [54] 2003	Gymnasts-Acrobatic/ artistic<br>Female	Case-control	N= 67<br>25 acrobatic gym<br>21 swim<br>21 controls	Years:<br>10 ± 0.7 acrobatic gym<br>11 ± 0.9 swim,<br>10.1 ± 1.1 controls	Gym: 1.5 yr training history, Gym 4.2 ± 1.7hrs/week	CA, weight, height, BMI, BF%, FFM	Tanner scale, stage 1 or 2, premenarche	Questionnaire: training & medical history, physical activities and Ca intake; EAT attitude test-26<br>Interview: dietary intake	Bone SOS, dom and non dom sides, distal 1/3 radius and midshaft tibia.	*Gym slightly shorter and lighter; BMI was sign higher in controls; BF% and SF's lower in athletes; FFM sign lower in gym<br>*No diff in Ca intake, below recommended<br>*Higher SOS in gym than controls (p=0.3); tibial SOS in gym & swim sign higher than controls (p<.001 for both)<br>*High correlation coefficients observed b/n dom and nondom sides of the tibias and radii<br>*Neither radius nor tibia SOS results correlated with any measures of body size
Kraan et al. [55] 2019	Gymnasts<br>Female and Male	Observational: exploratory study over 3 years	N= 66<br>27 symptomatic gym<br>16 asymptomatic gym<br>23 non gym	Median yrs:<br>14.5 symptomatic<br>14 non Symptomatic<br>13.3 non gym	Gym: 9 yr training history,<br>symptomatic gym 23hrs/week<br>asymptomatic 16hrs/week	CA, weight, height, BMI	Bone age	Evaluation of proximal physis of MC-1 (Kox et al.); MRI imaging of symptomatic wrist	Distal radial physis measured; changes in physeal volume vs difference in radial width.	*Median volume of the distal radial physis differed significantly between groups (p <.01)<br>*Gym with wrist pain suspected of having an injury of the distal radial physis- BA between 12 and 14 years<br>*The groups displayed no differences in medial-lateral distance of the distal radial physis (p = 0.59)<br>*Median volume of the proximal physis of MC-1 was sign different b/n symptomatic gym & non-gym (p <.01)
Kraan et al. [56] 2020	Gymnasts<br>Female and Male	Case-control	N = 69<br>27 gym,<br>18 asymptomatic ,<br>24 non-gym	Years:<br>14.4 ± 1.3 symptomatic,<br>14.1 ± 1.3 asymptomatic ,<br>13.6 ± 1.3 non-gym	Gym:<br>symptomatic 20.8 ± 7.0 hrs/week<br>asymptomatic 20 ± 10.8 hrs/week	CA, weight, height	Bone age	Questionnaire: biological information, sport participation, symptoms of wrist-physician PE, maximum grip strength test	Configuration of physes assessed by segmentation of cartilaginous part of the distal radial physis; by heat maps	*Thickness entire distal radial physeal surface was increased in both gym groups<br>*Ulnar border of the physis was the thinnest border in all gym<br>*Symptomatic gym increase in physeal thickness at volar part<br>*Distal radial physis in both symptomatic and asymptomatic gymnasts increased in all locations c/w controls<br>*Volar side widening most pronounced in symptomatic gymnasts

Alison S Fitch, Jocelyn Mara, Felicity Lord, Gordon Waddington. Corresponding author: Alison Fitch Email: sportsphysio@alifitch.com ali.fitch@canberra.edu.au Research Institute for Sport and Exercise, University of Canberra ACT 2617, Australia

Supplementary file S1. (continued)

Maimoun et al. [57] 2013	Gymnasts - Rhythmic Female	Case-control: 1 year follow up design	N= 72<br>24 rhythmic gym<br>24 swim<br>24 controls	Years: 10.8-18<br>Mean years: 14.2± 1.7	Gym:> 5 yr training history, 23.0 ± 2.7 hrs/week	CA, weight, height BMI.	Bone age, Tanner scale, Age at menarche	Training history collected and also a training recall diary covering previous 3 years	BMD(DXA); bone metabolism: blood samples	*Wt, BMI, and BMI percentile were sign lower in gym c/w control and swim; gym shorter than swimmers; FM lower in gym; BA and tanner stage delayed in gym<br>*Gym sign greater aBMD adjusted for age, FFM & FM c/w with others at femoral region (12.5- 14y, lasted to 18y);1 year follow up, sign increase at all bone sites for all groups<br>*annual aBMD gain decreased with age in each group; bone marker concentrations throughout growth decrease with age for all markers in every group
Nickols-Richardson et al. [58]1999	Gymnasts Female	Case-control: 1 year data collection	N= 18<br>9 artistic gym<br>9 non-gym as controls (soccer & martial arts)	Years: 8-13	From previous study on BMD & premenarcheal females, gym: 7.1 ± 0.6 yr training history, 15.7± 1.6hrs/week	CA, weight, height, FFM, FM, BF%	Premenarche	Dietary intake: 3-day record of daily energy; physical activity via 7-day recall + sleep + Daily energy expenditure	BMD, FM, FFM(DXA); rates of bone formation/ resorption: blood and urine samples.	*Gymnasts had sign lower FM (p<.05) and BF% (p <.01) at all measurement points c/w controls<br>*Gym sign higher BMD at all sites at all time points c/w controls<br>*1yr gym training: moderately larger % changes in trochanter , FN , LS and TB BMD<br>*Ca as a % of intake, decreased over 12 months (p<0.1), urinary cross-links sign decreased over 6 months in both groups
Scerpella et al. [59] 2016	Gymnasts Female	Observational: Prospective longitudinal	N= 44<br>Cohort 1: 20 (7gym/13 non gym),<br>Cohort 2: 24 (6 gym/18 non gym)	Years: premenarche up to 8 years post menarche	Gym: >2 yr training history, minimum 6hrs /week	CA, weight, height, BMI	Age at menarche - >gynage	Questionnaires assessed hours of physical activity per week	Bone properties; body composition (DXA)	*Average age menarche no sign dif, ht and LM similar between groups across maturity; gynage 1: gym sign more LM than non-gym,+6yrs non gym sign taller than gym (p<0.05)<br>*Gym reported injuries from gynage +3years onwards, 1/3 & ultra distal radius growth curves for bone area and mass were elevated in gym relative to NON<br>*FN growth curves> gym BMC & narrower gym endosteal diameter, similar periosteal width<br>*Lx gym BMC was > non gym BMC across growth (p<.02); subhead BMC av > gym than non-gym across growth (p<.036)<br>*gym slopes decreased post quit for ultra distal BMC, ultra distal IBS, femoral neck BMC, narrow neck cortical thickness and PA L3 BMC
Remmel et al. [60]2021	Gymnasts- Rhythmic Female	Observational: Prospective longitudinal study over 3 years	N= 89<br>45 rhythmic gym<br>44 untrained controls	Mean years: 8± 0.6 gym<br>8.2± 0.6 controls	Gym:10–14hrs/ week for the past 2 years	CA, weight, height	Tanner scale, premenarchal	Jump performance: max vertical ht, CMJ and rebound jumps with continuous CMJ's	BMC, FM and FFM(DXA); blood analysis	*From 8 years of age, gym had lower (p <.05) FM and leptin values<br>*Higher (p <.05) jumping performance measures c/w controls<br>*Gym more (p <.05) WB, FN and Lx BMC, respectively c/w controls<br>*WB, FN and Lx BMC increased more (p <.05) between 7 to 12 years of age in gym in c/w controls
Tournis et al. [61] 2010	Gymnasts- Rhythmic Female	Case-control	N= 60<br>30 gym<br>30 controls	Mean years: 11.26 gym<br>10.87 controls	Gym: training history > 2 years 2 sessions/day (~4.34± 0.25) 6 days/week.	CA, weight, height, BMI, BF%	Bone age, Tanner scale	Blood analysis	pQCT BMC left tibia; Biochemical analysis: fasting bloods (O/N) and 72h nil exercise.	*Athletes had sign lower wt, BMI, and SF sum.<br>*Gym had higher total Ca; iPTH lower in gym c/w controls<br>*Gym had higher total trabecular and cortical BMC and area, higher SSIp, higher muscle area<br>*+ve association between CA, ht, muscle area with cortical area, trabecular area, periosteal circumference, SSIp; but SF -ve association; at cortical sites: training age gym had +ve association with area, BMC, CRTHK; sCTX negative association with CRTHK and positive association with endocortical circumference<br>*P1NP positive association with trabecular CSA

Alison S Fitch, Jocelyn Mara, Felicity Lord, Gordon Waddington. Corresponding author: Alison Fitch Email: sportsphysio@alifitch.com ali.fitch@canberra.edu.au Research Institute for Sport and Exercise, University of Canberra ACT 2617, Australia

Supplementary file S1. (continued)

Ward et al. [62]<br>2005	Gymnasts<br>Male and Female	Baseline Data from RCT	N= 86<br>44 gymnasts<br>42 controls	Years:<br>9 gym<br>8.8 control	Gym: male 15hrs/week, females 14.7 hrs/week	CA, weight, standing height, sitting height	Bone age, Tanner scale, Testicular volume	Calcium intake: 3-day dietary recall food diaries , questionnaire for physical activity/week.	pQCT tibia, non dom side of forearm;<br>BMD(DXA)	*Gym shorter and lighter, BA not sign different<br>*Mean intake of Ca not sign different but greater than the UK reference for age group (800mg/day)<br>*Mid radius gym bone total area, periosteal circumference & cortical area sign greater with higher SSI than controls;<br>*Muscle cross sectional area was sign greater in gym:<br>*Male gym showed 13% greater thickness; female non-sign 3% smaller thickness<br>*Male gym muscle cross sectional area 11% larger than controls (1% b/n for females)<br>* Radius & tibia total VBMD was sign higher in both, and trabecular vBMD at the radius<br>*L1-L4, BMC, aBMD and BMD greater in gym; TB, BMC & aBMD greater gym
Caine et al. [114]<br>1992	Gymnasts<br>Male and Female	Observational case series	N= 60	Years:<br>9-17 female<br>9-18 male<br>Mean years:<br>12.65 female<br>12.56 male	Two national gym clubs (larger study): females 20-27hrs/ week males 7-25hrs/week.	CA	Bone age, Tanner scale	Training level (hrs/week) and competitive level established via clubs.		*5 gym had stress changes and BA was delayed in all cases<br>*4 of these female gym were in rapid growth during this period<br>*Males showed no sign difference between CA and BA<br>*Female sign delayed in maturation BA<CA
Corujeira et al. [63]<br>2012	Gymnasts-Acrobatic/ artistic<br>Female	Cross-sectional comparative analytical type study	N=27	Median years:<br>14.1 gym<br>15 controls<br>Median years:<br>11.8	Gym: median 18hrs/week	CA, weight, height, BMI, BMI z score	Bone age, Tanner scale, Age at menarche, PAH	BP, Food frequency questionnaire- Ca supply.	BMD (DXA)	*No sign diff in tanner stage or age at menarche<br>*Oligomenorrhea high prevalence in both groups<br>* Secondary amenorrhea occurred in 14% gym, absent in control<br>*No difference between groups for nutritional status, BMD, BA<br>*2/3 of gymnasts (67%) and 40% controls had a ht below 50th percentile<br>*PAH similar both groups (when based on BA, athlete has sign lower target ht)
Courteixi et al. [53]<br>1998 (B)	Gymnasts<br>Female	Cross-sectional comparison	N= 41	Mean years:<br>10.4 gym<br>10.5 swim<br>10.7 controls	Gym: 3yr training history in sports, NR for training hrs/week	CA, weight, height, BMI	Bone age, Tanner scale		BMD, BMC (DXA) Lx (L2-L4),non dom hip (FN, intertrochanter region, WT) and non dom radius.	*No sign diff between groups for CA, ht, wt, body composition, pubertal development; gym lower FM(kgs)<br>*No sign diff for BA but trend toward younger for gym<br>*FM correlated with WB BMC, WB BMD, FN BMC<br>*Lean tissue mass correlated with all BMC and BMD sites<br>*Lean tissue mass was the best predictor of BMC and BMD at all sites<br>*Slope OR line sign steeper in gym when assessing correlation between BMC/BMD and wt
DeSmet et al. [64]<br>1994	Gymnasts<br>Female	Cross-sectional	N= 201<br>Elite athlete & non athletes	Mean years:<br>15.9	Gym: mean 7.1yr training history, mean 27hrs/week	CA, weight, height	Bone age	Final score of the competition, anthropometric data and training history	Stress- related reactions; sclerosis (XRAYS)	* Gym and the nonathlete group ulnar variance was sign (p<.02)<br>*Ulnar variance and carpal angle were not related to CA, training intensity, or performance score in the competition<br>*Carpal angle was related to the age at which gym began their training (p <.05)<br>*Ulnar variance parameters correlated sign (p<.01) with ht and wt<br>*Stress-related changes of the radial physis were observed in 16 wrists (10%)

Alison S Fitch, Jocelyn Mara, Felicity Lord, Gordon Waddington. Corresponding author: Alison Fitch Email: sportsphysio@alifitch.com ali.fitch@canberra.edu.au Research Institute for Sport and Exercise, University of Canberra ACT 2617, Australia

Supplementary file S1. (continued)

**Jaffre et al. [65] 2003**	Gymnasts<br>Female	Case-control	N= 120<br>56 elite gym<br>64 controls	Years:<br>10.8 ± 1.7 gym<br>10.7 ± 1.7 controls	Gym: 12.4 ± 2.2 hrs/week	CA, weight, height	Bone age,<br>Tanner scale	Bone properties- bone speed of sound, broadband ultrasound attenuation for dom calcaneus	BMD(DXA)	*All subject's prepubescent<br>*Gym had higher BMD values at all skeletal sites except WB<br>*Gym sign higher BUA and SOS values.<br>*Results obtained after repositioning lead to a better discrimination of gym vs controls
**Troy et al. [66] 2018**	Gymnasts<br>Female	Prospective cohort, 4 years	N=22<br>10 gym<br>12 non gym	Years:<br>11.4-15	From a larger study: local gym clubs, 11.6 ± 4 hrs/week (other sports, hrs increased to 13.2 ± 5.1 hrs/week).	CA, weight, height	Tanner scale> gynage,<br>Age at menarche	pQCT images created for subjects; Loading stress: 1. axial compressive force 2. an off-axis force; both (1.6 X BW)	pQCT radius, BMC, BSI and CSI. Cortical measures of cross section and volume plus other bone structure ratio's	*At baseline, gym had stronger bone, including 26% higher BMC, 51% greater compressive strength, and 21% higher trabecular density<br>*Over the study period, both groups more than doubled their bone strength<br>*Pre-menarche principal stresses predicted change in pQCT variables for non-gym, but not gym<br>*Bone of non-gym became more asymmetrical than the bone of gym
**Võsoberg et al. [67] 2017**	Gymnasts-Rhythmic<br>Female	Observational longitudinal over 3 years	N= 50<br>25 gym<br>25 controls	Mean Years:<br>8 gym<br>8.2 controls	Gym: 6-14hrs/week (4-7 training sessions per week) for the past 2 years.	CA, weight, height, BMI	Bone age,<br>Tanner scale	Jump performance: max vertical height, 1x CMJ & rebound jump with continuous CMJ's for 15 secs.	BMD and body composition (DXA)	*Baseline age, BA and FFM were not different and increased similarly after 3 years<br>*Maturation slower in gym c/w controls, increase in ht & wt was lower in gym after 3 years<br>* Gym had lower BMI, BF% and FM at both measures, increase in BMI, BF% and FM over 3 years lower in gym<br>*WB BMD increased sign after 3 year study in both groups<br>*Baseline FN BMD higher in gym, FN BMD was higher in gym after 3 years<br>*Gym higher CMJ and RJ15s c/w controls at both measurements<br>*Gym only FFM increase correlated with BMD at baseline, baseline RJ15s most sign predictor of change FN BMD
**Amorim et al. [68] 2017**	Dancers<br>Female	Cross-sectional: matched paired study	N= 64<br>34 dancers<br>30 controls	Years:<br>10.9± 0.7 dancers<br>11.1± 0.5 controls	Dancers: Training history first year students,<br>Dance training consists of 2-4 classes a day	CA, weight, height	Tanner scale,<br>Age at menarche	Nutrition via 3-day food diary; calcium intake.	BMD (DXA)<br>Dances used (GE Lunar Prodigy)<br>Controls used (Holigic)	*Dancers had lower wt and ht, higher numbers at tanner stage 1(67.6%)(controls stage IV, 40%)<br>*Dancers had greater Ca intake (p=.03)<br>*Dancers lower(p<.001) crude BMD and BMC to controls on all sites; significant lower BMAD by 43.6% on dancers c/w controls (p<.001) at FN and 17.6% lower at Lx(p<.001)<br>*When bone parameters adjusted for wt, ht, Tanner stage and Ca intake dancers still had sign lower BMD at forearm and FN<br>*BMAD values at FN were also sign lower in dancers than controls(p<.001)
**Sommella et al. [69] 2019**	Dancers-Ballet<br>Female and Male	Prospective cohort study over 3 years	N=10<br>5 females<br>5 males	Years:<br>12.4 ± 0.38	Dancers from Royal Ballet School (from previous cross sectional study), 23hrs/week	CA, weight, height, BMI	Weight, height in real time	Sit and reach test, flexibility, Beighton's score, flamingo balance test, plank, ankle strength PF's	BMD, body composition (DXA); 3D motion analysis system: Lumbar spinal motion	*Sign improvements in weight, height, BMI, BMD total, BMC, BMD legs and arm and LM; small increase in z score<br>*Flexibility improved after 3 years in flexion Lx (p<.025), left side lateral flexion improved<br>*Sign increase in flexibility by SART and plank test<br>*Ankle PF strength improved<br>*Sign correlations between change BMD and power right PF; the change in BMC left and change power both PF's and change in BMC left and change in isometric PF

Supplementary file S1. (continued)

**Young et al. [70] 1994**	Dancers-Ballet Female	Case-control	N= 85<br>44 dancers<br>23 healthy controls<br>18 amenorrheic	Mean years:<br>17 dancers<br>18.1 healthy controls<br>16.7 amenorrheic	Dancers: > 32hrs/ week.	CA, weight, height	Age of menarche		BMD, body composition (DXA)	*Dancers weighed more than girls with anorexia nervosa but less then healthy controls<br>*Total BMD of dancers similar to health controls but higher anorexia<br>*WtB sites dancers BMD similar to controls and higher anorexia<br>*Non WtB sites dancers had similar BMD anorexia but lower than controls<br>*When adjusting BMD for wt, dancers had higher BMD then controls at WB sites<br>*When adjusting for age, BMD at WtB sites, higher dancers and reduced at NWB sites, with anorexia was reduced at all sites
**Burckhardt et al. [71] 2011**	Dancers-Ballet Female	Cross-sectional over 2 consecutive years	N=127	Years:<br>15-18	Pre-professional student dancers: 22.1 ± 7.6 hrs/week dancing.	CA, weight, standing height, BMI	Tanner scale, Age at menarche	Nutritional status (EAT-40 questionnaire) and qualitative 3-day dietary record	BMC, BMAD (DXA) FN and Lx (L1-L4)	*Menarche was late; 42.5% normal BMI, 41.7% grade 1 thinness, 12.6% grade 2 thinness, 6.7% grade 3 thinness<br>*10 had primary amenorrhea, 1 had secondary amenorrhea<br>*Lx BMAD was 37% below 5th %; FN BMAD sign higher than reference population<br>*Lx BMC: age, wt, ht, BMI, yrs since menarche, dairy intake positive association<br>*Lx BMAD: age, yrs since menarche positive association, non-dairy proteins intake negative association<br>*FN BMC: wt, ht, BMI, dancing hrs/wk dairy intake positive association, non-dairy protein intake negative association<br>* FN BMAD: wt, BMI, age start dancing, dairy intake positive association
Injury, Illness and Pain									
**Sweeney et al. [79] 2019**	Gymnasts Female	Cross-sectional	N=29<br>11 gym back pain<br>18 gym without back pain	Mean years:<br>Back pain 13.3<br>No back pain 13.8	Gym :<br>LBP affecting 24.2 ± 4.8 hrs/week<br>LBP not affecting 21.7 ± 5.7 hrs/week	CA, weight, height	Age at menarche	LBP - continued during gym/activities/ADLS(Micheli Functional Scale and Oswestry Low Back Pain Disability Questionnaire)	Activity level of gym passive and active flexibility	*No sign difference in flexibility measures and LBP in this population<br>*11/29 reported LBP affected gym activities; no sign difference in participant characteristics or gym activity level between groups<br>*A sign > proportion of gym with LBP that affected their gym activities reported pain with jumping, relative to gym whose LBP did not affect their participation in gymnastics activities<br>*A sign> proportion of gym with LBP that affected gym reported pain with heavy lifting weight
**Schevchenko et al. [80] 2008**	Gymnasts and Trampoline Female	Cross -sectional	N= 175<br>130 athletes<br>45 controls	Years:<br>10-17	Gym:<br>18-20 hrs/week	CA, weight, height, leg length , shoulder width, four pelvic, body ratios's calculated, BMI, BF%	Puberty scale, Age at menarche	Morphological and anthropometric characteristics for prediction of hormonal health and general health	Clinical assessments. observed via ENT, ophthalmologist, ,endocrinologist and gynaecologist	*Gym lower ht, wt, BF% lower; BF% decreased with age in gym and below normal for biological development; shoulder to hip ratio higher in gym<br>*Gym highest ratio leg length/ht (strong indicator of hypo-estrogenenia), pelvic indices < 55 indirectly suggest hormonal imbalance<br>* Menarche delayed 1.5yrs in gym, serious delays with gym with 16% more than 4ys, 63% at 2yrs<br>*Gym higher % of mums who had delayed menarche and had trained<br>* Gym started training 4- 6yrs and intense training(18-20h/week) and more childhood diseases than control groups (infection index > 4), chronic sub-acute tonsilitis >50%
**Rudavsky et al. [81] 2017**	Dancers Male and Female	Cross-sectional	N= 68<br>60 adolescent students<br>8 mature adults	Years:<br>11-18 adolescent students<br>21-40 mature adults	ABS, Victorian College of the Arts: NR hours of training	CA, weight, height, sitting height	PHV (pre, per, post PHV)<br>Older group-skeletally mature.	Patellar tendon formation: UTC	Categorise appearances of tendon attachments on greyscale imaging in all planes.	* AP thickness increased by group PHV at 1cm and 2cm from inferior pole of patella (p=.001 and p=.007 respectively)<br>*Arbitrary grouping increased with skeletal maturity (p=.024); lower maturity (pre and peri -PHV) distribution of greyscale scores, higher maturity offset (post PHV) and mature group participants were in greyscale iii<br>* Pre and peri PHV had greater variability of echo pattern, variance decreased sign in post and mature groups for the % of echo type i<br>* a trend of increased echo type i and decreasing % of echo-type ii across skeletal maturity

Supplementary file S1. (continued)

**Hamilton et al. [82] 1997**	Dancers<br>Female	Longitudinal,<br>4 year sample period	N= 40<br>elite dance students	Mean years:<br>14.92	School of American Ballet:<br>17hrs/ week dance	CA, weight	Tanner scale,<br>Age at Menarche	Injury history and physical examination for ROM, dance skills, asymmetries;<br>Boney morphology<br>Menstrual health	Psychological traits: self-image questionnaire;<br>Nutrition intake, eating problems: 2-day diet history (two 24hr recalls): EAT-26	*33% had scoliosis curves, 83% had been injured in ballet class, as the year progressed, 64% developed additional injuries~54 days duration<br>*dancers who developed minor injuries had > anatomical problems and dynamic deficits on initial orthopaedic exam than the uninjured dancers<br>* Average age of menarche was delayed (13.88 vs 12.5 yrs), 15% had yet to reach puberty and 36% had secondary amenorrhea<br>*10% of dancers developed stress fractures, those that incurred overuse injuries were more bulimic (EAT-26 1.65 vs 0.22, p<.004)<br>*Food intake 87% RDA for calories, 82% for calcium, 16% < ideal wt for ht<br>* eating disordered students had marked asymmetries in leg length, flexibility (recurvatum), and turnout (knee), > missed classes due to injuries
**Rudavsky et al. [83] 2018**	Dancers<br>Male and Female	Prospective longitudinal	N=57	Years:<br>11-18	ABS, Victorian college of the Arts: 4-8 hrs/ daily.	CA, weight, height, sitting height	PHV	Tendon health/ pathology and pain	Training load	*Normal group: 1 pre-PHV by end study, 8 post-PHV end study<br>*Pathology group: 1 pre-PHV and 2 post PHV entire time<br>*5/57 dancers developed hypoechoic areas at least 2 time periods and were considered to have tendon pathology<br>*Difference in type iii and iv disorganized tissue between the groups through study (p = 0.001 type iii, p =.01 for type iv)<br>*By end study,4th time period, increased disorganized echo-type iii+iv, combined, of 4.1% for the whole cohort (p <.01); abnormal group showed greater increase of 13.6% more type iii+iv<br>* pain trended higher during the study in the pathology group (P =.055), 3 pathology group participants had no pain on the SLDS during the study while two reported moderate knee pain (the same 2 participants that developed pain on the VISA-P)
**Steinberg et al. [84] 2020**	Dancers<br>Female	Cross-sectional	N=71	Years:<br>12-15	Dancers:<br>15.3 ± 4.2hrs/week	CA, weight, height, BMI	Tanner scale,<br>Age at menarche	Achilles tendon structural development (UTC)	Dance history and intensity of training	* No sign differences between pre and post menarche dancers in % dancers with pain in Achilles tendon (VAS >=3); impact of training lower among pre-menarche group; age, wt, ht, BMI lower in pre-menarche group<br>* Dancers post menarche had a sign lower prevalence of echo type i fibres and sign > prevalence of echo type ii and iii and iv fires in right and left legs<br>* Post menarche dancers had sign > cross-sectional area in both left and right Achilles tendons c/w pre-menarche dancers.<br>* Post menarche dancers, increased BMI percentile correlated with decreased echo type I and increased echo type ii, iii, iv<br>* Effect of menarche was sign (p=.023) on type ii fibres
**Steinberg et al. [85] 2012**	Dancers<br>Female	Cross-sectional	N=1336	Years:<br>8-16	Dancers:<br>3-11hrs/week	CA, weight, height, BMI	Age at menarche	Anatomical anomalies, Joint range of motion, dance technique	Injuries	* 42.6% of dancers had an injury sign association between dance practice in specific position and injury<br>*Association between early age of onset of menarche (<12yrs) and rate of injuries (p=.04)<br>* Most dancers with back injury had hypo hip ER ROM<br>*non-categorised injuries manifested in hyper hip ER ROM<br>* Scoliosis showed a sign association with injury, dancers 8-12 with scoliosis was 1.62 x > c/w dancers without scoliosis, and 1.52 x> c/w adolescent (13-16) dancers; scoliosis had back injuries and knee injuries, non-scoliosis group had knee injuries and non-categorised injuries<br>* Ankle PF, hip abduction and age important in predicting knee injury; dancing time and hip abduction predicting foot or ankle tendinopathy ; scoliosis, ankle PF and dance technique(rolling in) predicting back injuries; non-categorised injuries predicted by dancing time, ankle PF, hip abduction and dance technique (rolling in) and age

Alison S Fitch, Jocelyn Mara, Felicity Lord, Gordon Waddington. Corresponding author: Alison Fitch Email: sportsphysio@alifitch.com ali.fitch@canberra.edu.au Research Institute for Sport and Exercise, University of Canberra ACT 2617, Australia

Supplementary file S1. (continued)

**Ziegler et al. [86]**<br>**1998**	Ice skaters<br>Female	Cross-sectional	N= 21	Mean Years:<br>13.7 yr (11-16)	Indiana World Skating Academy and Research Centre<br>Training hours not reported	CA, weight, height, BF%, BMI	Age at menarche	Training, Menstrual regularity	The eating attitudes test relation to diet and weight control, 3 day food record and analysis; iron status	* Estimated energy intakes were below RDA and intakes of iron and calcium low<br>* Individual EAT scores failed to predict energy intakes<br>*EAT scores correlated weakly with calcium, thiamine, and riboflavin intake<br>* No statistically significant relationship was found between menstrual status (either regular, irregular, or never menstruated) and BF%
**Steinberg et al. [72]**<br>**2025 B**	Gymnasts- Rhythmic, acrobatic and artistic<br>Female	Cross-sectional	N= 274<br>154 rhythmic<br>60 acrobatic<br>60 artistic	Years:<br>11.8+/- 1.9<br>2 groups: Tanner stage ≤2 and ≥3	Gym:<br>> 4 national/ international comps/year, mean 23.1 hrs/week (current yr)	CA, weight, height, BMI, BMI%, BF%	Tanner scale, Age at menarche	PFP, ROM, hypermobility, muscle strength	Bone density, Training load	*21.5% of gym had PFP; no sign difference in prevalence of PFP in gym with and without menarche or pre/post pubertal Tanner stage (≤2 , ≥3) (p> 0.05)<br>*Sign did b/n prevalence of gym with and without PFP in the 3 disciplines (p=0.008, prevalence of PFP in the artistic gym (33.3%) sign higher than in acrobatic (11.7%) (p=0.012). No sign difference seen in the prevalence of PFP in gym with and without menarche, and in those pre/post pubertal Tanner stage (<=2 , >= 3) (p> 0.05)<br>* Gymnasts with PFP had sign higher h/week training over the past year and h/week during the current year c/w to gym without PFP (p=0.010 and p= 0.008 resp)<br>* Tibial bone density was sign different b/n gym in disciplines (p= 0.005) with sign lower density in gym with PFP compared to gym without PFP (p=0.047)<br>* mm strength were sign lower and ROM sign higher in gym with PFP cw gym without PFP (sign higher in acrobatic group-strength; sign lower artistic-ROM)
**Pentidis et al. [73]**<br>**2021**	Gymnasts-Artistic<br>Female<br>Male	Cohort-longitudinal<br>12 months	N= 32<br>21 gym<br>11 controls	Years:<br>Gym: 9.2±1.7	Gym:<br>22.5 hrs/week	CA, height, weight, BMI, tibia length	Tanner scale	Muscle-tendon properties, strength	Ankle ROM, Training	*Imbalanced adaptation of muscle strength and tendon stiffness together with > fluctuations of muscle strength gave > tendon strain fluctuations over 1 yr (P = 0.017) and higher frequency of athletes with high-level tendon strain (≥9%) cw nonathletes.<br>*Greater mm strength (p<0.001) in athletes cw non athletes , no difference in Achilles tendon stiffness (p=0.252), indicating a training induced imbalanced adaptation of mm strength and tendon stiffness in athletes<br>*The maximum ankle joint moment was significantly greater in athletes compared with nonathletes (P < 0.001)
**Lye et al. [74]**<br>**2024**	Dance-Ballet<br>Female	Cross-sectional	N=89<br>48 ballet<br>41 controls (mixed sport cohort)	Years:<br>13-18	All athletes minimum international level<br>Ballet:<br>12hrs/week	CA, height, weight, BMI	Menarche (post menarche ballet dancers)	LEA (LEAF-Q)<br>ED (BEDA- Q)	Training	* adolescent athletes and ballet dancers had a similar prevalence of being at risk of LEA (61.98% versus 54.17%, respectively, p = 0.52)<br>*Age had positive correlation with LEAF-Q scores (p < 0.05),as age increased, the risk of LEA also increased (dancers and controls)<br>*Training hours showed a negative correlation with LEAF-Q scores p < 0.01, i.e. as training hours decreased, the risk of low energy availability increased.<br>*Fewer athletes (39.02%) were considered at "low LEA risk" cw dancers (45.83%)<br>*Dancers classified as "low LEA risk", (63.6%) 15 years old or younger<br>* "at risk of ED" absent in 90.2% both adolescent athlete's and dancers, LEA likely unintentional nature

Alison S Fitch, Jocelyn Mara, Felicity Lord, Gordon Waddington. Corresponding author: Alison Fitch Email: sportsphysio@alifitch.com ali.fitch@canberra.edu.au Research Institute for Sport and Exercise, University of Canberra ACT 2617, Australia

Supplementary file S1. (continued)

**Everaert et al. [75] 2024**	Dance-<br>Ballet, modern, contemporary<br>Female	Cross-sectional<br>12 months<br>Retrospective on injury data	N=50	Years:<br>15.9± 1.26	Training dance years:<br>9.4 ±2.84<br>Pre-professional dancers<br>20.3hrs/week	CA, height, weight, BMI	Bone age,<br>Age at menarche	LBP(NMQ-E)<br>ROM, scoliosis, hypermobility	Bone maturation,<br>Menstrual health	* BA sign lower cw CA in both the LBP group and the no LBP group (p<0.05)<br>* Delayed bone maturation in 32%<br>* 53% dancers had irregular menstrual cycle or amenorrhea during past 6 months<br>* Dancers with LBP started dance activities 2 years earlier than dancers without a history of LBP and showed sign less Internal left hip ROM (p<0.05); sign difference between total left hip ROM cw total right hip ROM in LBP group, not present in no LBP group<br>* dancers with scoliosis, GJH, delayed bone maturation and aberrant menstruation did not differ between those with and without LBP
**Donti et al. [76] 2025**	Gymnastics-<br>Artistic<br>Female	Cross-sectional	N=84<br>39 gym high level<br>45 recreational	Years:<br>12-18	High level gym:<br>22.5hrs/week	CA, height, weight, BMI	Age at menarche	ED(EDE-Q)<br>REDs knowledge	Menstrual health,<br>Training characteristics	*High-level artistic gym more training experience, lower weight, BMI than low-level artistic gym(p < 0.05);scored higher in EDE-Q global score cw low level gym and (p < 0.05)<br>* 58.3%concerned amount of food (EDE13);57.2% lost control of eating min x1 (EDE14),50% min x 1 vomiting after binge eating (EDE16), 39.3% use excessive training as avoid feeling guilty about eating (EDE18)<br>*On average, 51.5% of artistic gym provided incorrect answers related to REDs<br>*more high-level artistic gym missed menstrual cycles in the last 3–4 months (p < 0.001)
**Steinberg et al. [77] 2024**	Dance-<br>Classical and modern ballet,<br>Jazz<br>Female	Cross-sectional	N=49	Years:<br>13.6±2.9	Dancers:<br>Mean<br>19.55hrs/week	CA, height, weight, BMI	Tanner scale,<br>Age at menarche	PFP,<br>Tendon structure and<br>Bone properties	Joint ROM,<br>Strength, VAS pain,<br>Training intensity	*PFP was found in 49% of the participants<br>*sign reduced radius and tibial bone properties (SOS) in dancers with PFP cw no PFP<br>*Post pubertal with no PFP > strength, tendon structure cw pre- pubertal with PFP and pre pubertal with no PFP<br>*dancers with PFP had> en pointe ROM(p < .001), > hip ER ROM (p < .001) cw no PFP<br>*Post-pubertal dancers sign higher all anthropometric measurements (p < .001), > patellar thickness (p = .033) cw pre- pubertal dancers
**Little et al. [78] 2023**	Gymnastics<br>Female<br>Male	Cross-sectional	N=73<br>11 worried wt<br>62 not worried wt	Years:<br>Worried<br>13.3 ± 2.9<br>Not worried<br>10.9 ±2.8	Gym:<br>Mean<br>15.35hrs/week	Ca, height, weight, BMI	Age at menarche	Disordered eating (questionnaire),<br>Generalised anxiety disorder (GAD-7)	Bone health,<br>Menstrual health,<br>Training characteristics	*15% (n=11) who worrying about their weight, sign older and taller cw who didn't worry<br>* worrying about their weight had sign higher GAD-7 scores cw who didn't worry<br>* sign higher worrying about their weight also thought they were fat when others say they are thin and had experienced menarche cw who did not worry about their weight<br>*Sex, competition type, BMI, BMI-for-age percentile, training volume were not sign different b/n groups.<br>*Adjusting for independent effect of age, worrying about weight was no longer sign associated with GAD-7 scores (p=0.10), beliefs about their body size (p=0.07), or menarche (p=0.44)
Anthropometric										
**Baydar et al. [96] 2007**	Gymnasts<br>Female	Observational case matched study	N= 21<br>12 gym<br>9 controls	Mean years:<br>Gym 10.1± 0.99	Gym:<br>5 year history, trained 4-5 days/week<br>(12-15hrs)	CA, height, weight, BMI, skinfolds, BF%	Pre puberty-<br>(Pre- menarche)	Dietary intake: 7 day consecutive food record	Blood samples:<br>Lipid profile TC, LDL, HDL and TG	*Daily energy intake was met, Cho intake was ~50% in both groups, gym daily fat intake (5% lower) and protein intake (8% higher) c/w controls<br>*Lipid profile showed higher HDL (p<.01) and lower total Cholesterol , total TG, LDL and VLDL (p<.01)in gym c/w controls<br>*Gym showed lower wt (27.4/32.5), BMI (14.26/17.5) and BF% (17.8/22.8)c/w age match controls.<br>* Gym had sign lower wt c/w age and ht matched controls (p<.01), BF% was sign lower in gymnasts versus controls (p<.01)<br>*Gym had higher % of FFM (p<.01) than reference group

Alison S Fitch, Jocelyn Mara, Felicity Lord, Gordon Waddington. Corresponding author: Alison Fitch Email: sportsphysio@alifitch.com ali.fitch@canberra.edu.au Research Institute for Sport and Exercise, University of Canberra ACT 2617, Australia

Supplementary file S1. (continued)

**Camargo et al. [97] 2014**	Gymnasts- Rhythmic female	Cross-sectional 2months Oct/Nov 2012	N= 136	Years:<br>Gym: 9-16years (12.3± 1.9 years).	Gym:<br>5.4 ± 2.3 year history.<br>15-24hrs/week	CA- four age categories. 9-10yrs, 11-12ys,13-15yrs, 15yrs above; weight, height, sitting height, skinfolds, BF%	Age at menarche, PHV	Training hours questionnaire		*Age at menarche: 11-15yrs (mean 13.2± 1.3yrs)<br>*Gym weight below ref standards<br>*Height below WHO reference for all ages but below mean until 12 years but above mean from 13 years to Brazilian reference<br>*PHV occurred at 12.1 ± 0.8 years<br>*Body composition at PHV (0) were sign different from other somatic maturity groups (−3 to 5), except for standing height, weight and FFM (not different group 1); for sitting height (not different group −1); BF% (not different groups −2 to 2 and group 5) and FM(not different groups -1 and 1)
**Kutac et al. [98] 2019**	Gymnasts Female	Case-control	N= 668<br>16 Gym<br>652 controls	Years:<br>Gym and controls 8-12	Gym: 5 year training history,<br>5 ± 0.8 training days/ week and 3.7 ± 0.5 training hours day	CA, weight, height, BMI, body fat, total body water, FFM, MM, visceral fat		Determination of mean age for gymnasts, Normalisation index (Ni).		* Gym in the youngest competition category already differ in basic anthropometric parameters from the general population<br>* From 9years, gym have lower ht (except for one person) and wt than girls in the general population, ht and wt are below average or highly below average in nine gymnasts (56.3%)<br>* The high volume of specific physical activity of the gym, included in their training, affects their body composition parameters<br>* The gymnasts BF% and visceral fat values are below average to highly below average, and higher skeletal muscle mass (%), with values above average or highly above average
**Abalo-Núñez et al. [99] 2018**	Gymnasts- Aerobic Female and Male	Observational: Quasi-experimental retrospective and longitudinal study<br>1 year data collection	N=73 athletes<br>51 aerobic gym (M=6, F=45)<br>controls (F only)	Years:<br>Gym: 13.61(SD 4.59)<br>Controls:<br>14.59 (SD 3.93)	Gym: International competitors 6.85± 0.4 days/week; national 4.83± 1.5 days/week; regional 3 days/week.	CA, weight, height		Q angle, Weight bearing right and left legs, thigh perimeter right and left legs	Questionnaire for injury collection	* Previous injury increased risk of injury:<br>26 gym with no previous injury, 2 got injured during season<br>25 gym with previous injury, 10 reinjured during season<br>*Higher risk of injury if training 7 days a week: all injured in this group trained more than 2 hrs/day.<br>*Left Q angle had biggest influence on LL injuries – depends on gymnast weight<br>* Weight imbalance i.e. uneven b/n left, and right legs can influence injury in athletes
**Erlandson et al. [100] 2008**	Gymnasts Female and Male	Observational: 3-year mixed longitudinal study with one-time follow up measure	N= 453<br>231 Male<br>222 Female	Years:<br>9-18	Gym:<br>Mean 12.7-16.5 hrs/week	CA, weight, height, sitting height; mothers and fathers heights for PAH; age groups standardized into 12-month groupings for age	Tanner scale, Age at menarche, continuous measure biological age, PAH	Data from 3yr mixed longitudinal study, plus 1 year follow up for adult stature;<br>five age groups	Average weekly training hours;<br>Follow up: questionnaire at 10yrs	* Gym age of menarche at sign older age (p<.05), mothers of gym had sign older age at menarche (p<.05)<br>*When compared to standard growth curves, gym were below average height (50th%) at all CA during growth period up to 16years<br>*Target heights were significantly shorter In gym than predicted target heights of tennis players and swimmers (p<.05)<br>* Gym reached tanner stage 5 breast(B5) & pubic hair(PH) at older CA, when tanner was related to years from menarche, no sign difference in biological age entry into B5 or PH5<br>* No sign difference between adult heights in 3 groups (p>.05) or between those that retired earlier in adolescence and those that continued in sport

Alison S Fitch, Jocelyn Mara, Felicity Lord, Gordon Waddington. Corresponding author: Alison Fitch Email: sportsphysio@alifitch.com ali.fitch@canberra.edu.au Research Institute for Sport and Exercise, University of Canberra ACT 2617, Australia

Supplementary file S1. (continued)

**Weimann et al. [101]2000**	Gymnasts<br>Female and Male	Cross-sectional	N= 40<br>22 female<br>18 male	Years:<br>Female 13.6± 1 years<br>Male 12.4 ± 1.6 years	Gym:<br>Female: 22.1 ± 1.7 hrs/week<br>Males: 15.9 ± 5.0 hrs/ week	CA, weight, height, skin folds, body composition.	Pubertal clinical scale,<br>Hormone levels to ascertained info about the gonadal adrenal and hypophyseal axes.	Energy intake: recorded and assessed over 3 days including one day without training.<br>Bloods for other serum parameters: eg iron, ferritin	Training level	* Female gym ht and wt below 12th percentile of German adolescent women; BA (11.9± 1.5) compared to CA (13.6 ± 1)<br>*Male gym ht and wt 90th percentile of German adolescent man; CA and BA near identical, 12 ± 1.5years.<br>*Female gym trained more than males during puberty<br>*Moderate to extreme delayed BA in female gym; male corresponded to CA; PAH below calculated familial target height in female gym but not males<br>* Females; oestrogen, and oestradiol correlated with wt; no sign in oestrogen level in prepuberty to pubertal groups; Male: sign increase in pubertal rise in testosterone level<br>*DHEAS sign increased in both sexes during puberty<br>*FM and FFM between prepubertal and pubertal gym were sign different for all female gym, males only BMI and FFM were; female FM increased in relation to wt, males FFM increased in relation to wt; male FM was lower in pubertal, female FM was higher; males developed higher muscle mass during puberty<br>* Female gym showed an average nutritional intake below 50% for vitamin A, vitamin B complex, vitamin D, magnesium, calcium, and iodine and <RDA<br>*Male elite gym ~ 50% or greater for vitamin A, vitamin D, iodine, and carbohydrates
**Steinberg et al. [102] 2017**	Dancers<br>Female	Case-control	N= 542<br>271 with PFPS<br>271 without PFPS	Years:<br>PFPS 10-16<br>Without PFPS 10-16years matched.<br>34 dancers (10-11yrs), 120 dancers (12-14years) and 117 dancers were premature dancers (15-16years).	Pre-professional dancers:<br>9.84 ± 4.52	CA, height, weight, BMI		Knee stability;<br>Range of motion parameters:<br>patella, lower limb, hip, back and scoliosis	Training /week	* A trend was found among the patellofemoral pain syndrome (PFPS) dancers of greater prevalence of mobile patella<br>Factors associated with PFPS:<br>* 10-11 yr olds showed sign > hip abduction and limited lumbar and hamstring range of motion (ROM)<br>* 12-14year olds had sign shorter foot length, > ankle dorsiflexion (DF) ROM and knee flexion ROM and > prevalence of hind-foot varum and scoliosis and > prevalence of hypermobility patella in extended knee and with patella at 30deg flexion<br>* 15-16year olds had sign lower plantarflexion (PF) ROM, greater knee flexion ROM, lower hip internal rotation and greater prevalence of scoliosis<br>* Regardless of dancers age, higher % of anatomical anomalies (scoliosis and hind-foot varum), and different joint range of motion (lower ankle PF, greater ankle DF and greater knee flexion ROM) were found among the PFPS group c/w controls.
**Steinberg et al. [94] 2025 A**	Gymnasts-<br>Rhythmic, acrobatic and artistic<br>Female	Cohort (1 data capture)	N= 274<br>154 rhythmic<br>60 acrobatic<br>60 artistic	Years:<br>11.8 ± 1.9<br>2 groups: young 9-12years and adolescent =>13years	Gym:<br>> 4 national/ international comps/year, mean 21.65 hrs/week (current yr)	CA, weight, height, BMI, BMI%	BA ><br>predicted height,<br>Tanner scale,<br>Age at menarche	Training/week	Bone properties(SOS), radial and tibial strength	* In both age groups, artistic gym significantly higher BMI percentiles than rhythmic gym(P<0.05)<br>*In adolescent group, final-height prediction for rhythmic gym was sign greater than artistic gym(P<0.05) and BA was lower than chronological age (adolescent) (P<0.05)<br>* Greater tibial bone-strength, higher final-height prediction in rhythmic and acrobatic gymnasts compared cw artistic gym(P<0.05)<br>*sign age-group effect for training impact (age gym began their training, training hours/wk for current and previous year), anthropometric parameters, and bone properties (P<0.5)<br>* Artistic gym shorter than rhythmic and acrobatic gym and age at menarche later<br>* Despite similar BMI, BF%, maturity patterns, and training-volume history, artistic gym had lower bone-strength than rhythmic and acrobatic gym

Alison S Fitch, Jocelyn Mara, Felicity Lord, Gordon Waddington. Corresponding author: Alison Fitch Email: sportsphysio@alifitch.com ali.fitch@canberra.edu.au Research Institute for Sport and Exercise, University of Canberra ACT 2617, Australia

Supplementary file S1. (continued)

Liu et al. [95] 2024	Dancers-<br>Dance sport<br>Chinese dance<br>Female	Cross-sectional	N=131	Years:<br>16±1.3	Dancers training history:<br>2.9±1.8years,<br>19.5±<br>10.7hrs/week	CA, height, weight, BMI, BF%	Age at menarche	Body composition BF%	Training hours<br>Menstrual health	* Average BF% was 22.6±3.0%, 51 dancers (38.6%) showed lower BF%<br>*Average BMI of the dancers was 19.4±2.2kg/m2, and 47 (35.6%), 79 (59.8%), 5 (3.8%) dancers were underweight, normal, and overweight BMI respectively<br>* Average age of menarche was 12.5±1.1 years, menstrual cycles/year 10.5±2.7<br>* Twenty-nine dancers (22.1%) had secondary amenorrhea., 8/29 dancers <5 periods/yr<br>* Secondary amenorrhea group had lower BF% than the normal group
Biomechanical										
Burt et al. [89] 2010	Gymnasts<br>Female	Observational	N= 25<br>Pre-pubertal girls	Years:<br>7-13	Gym:<br>International average of 26.42 ± 3.86 hrs/week, age-matched nationals<br>13.85±2.64 hrs/week	CA, weight, height, sitting height, weight, BMI	Tanner scale	Wrist and ankle GRF's in skilled and less skilled	Movement patterns in less skilled and more skilled	* International gym had increased training hrs and for the same duration, had higher frequency of observed gym specific movements.<br>*International gym executed a higher frequency of rotations compared with national gym<br>*International gym had more refined training program<br>* Participation level appeared to have the strongest influence on observed variability in skills involving rotations and wrist impacts<br>* Ground reaction forces associated with national level gym skills were lower than those reported for international level gym skills.<br>*Between group differences were not evident in performing fundamental gym skills for higher and less skilled gym
Steinberg et al. [90] 2018	Dancers<br>Female	Prospective cohort study	N= 67 dancers	Years:<br>Grade 7<br>(12.8 ± 0.5)	Dancers:<br>6 years training history, mean<br>12.0 ± 3.1 hrs/week	Weight, height, BMI	Age at menarche	Passive joint ROM, training history	Beighton hyper mobility test	*Sign increase in most anthropometric parameters (p>.05), all parameters measured the intensity of training showed a significant increase from Grade 7 to Grade 8 (p <.05)<br>*En-pointe, sign increased joint ROM in Grade 8 compared with Grade 7 for all three menarche groups of dancers (p <.05) and in Grade 8, the No-menarche group manifested significantly greater En-pointe ROM compared with the Yes menarche group<br>*Hip ER (p =.007), ankle-foot En-pointe (p =.007) and ER:IR ratio (p =.011) were found to sign correlate with hours of practice in all three menarche groups
Bowerman et al. [91] 2014	Dancers<br>Female and Male	Observational prospective 6month study	N= 46<br>adolescent ballet dancers	Years:<br>16 ± 1.58	ABS FT students. Not reported hrs/week training	CA, weight, height,	Tanner scale, Age at Menarche	Lower extremity alignment during dance movement, foot length	Overuse Injury	* Age, ht, wt, maturation level and menstruation were all linked to trivial observed changes in injury risk- these effects unclear.<br>*59 injuries reported for 29 dancers over 6 months; 16 dancers suffered multiple injuries; feet had highest number of injuries; majority of injuries were S1 (modified)<br>* Right knee angles, pelvic angles, changes in foot length were associated with clear changes in injury risk (r foot length change 0.5 cm was linked to moderate increase injury risk (RR= 1.41, CI= 0.93-2.13)<br>* A 10 degree greater right knee angle (improved alignment) in both the fondu and temps levé resulted moderate decrease in injury risk for the fondu(RR= 0.68, CI= 0.45-1.03) and small decrease in injury risk for the temps levè (RR= 0.72, CI = 0.53-0.98)<br>* A 2 degree greater pelvic angle (poor alignment) for the temps levè on the L leg was associated with a moderate decrease in injury risk (RR= 0.52, CI = 0.3- 0.9)<br>*A 2 degree greater pelvic angle for the fondu on the R leg was associated with a small increase in injury risk (RR= 1.28, CI = 0.91-1.80)

Alison S Fitch, Jocelyn Mara, Felicity Lord, Gordon Waddington. Corresponding author: Alison Fitch Email: sportsphysio@alifitch.com ali.fitch@canberra.edu.au Research Institute for Sport and Exercise, University of Canberra ACT 2617, Australia

Supplementary file S1. (continued)

**Longworth et al. [92] 2014**	Dancers<br>Female	Cross-sectional ; matched pair study	N= 60<br>30 dancers<br>30 non dancers	Years:<br>9-16 (mean 12)	Dancers: min 3 years dance experience and > 4hrs structured dance training with qualified instructor per week; 6.13± 2 dance plus practice of 1.2± 1.1 /week	CA, weight, height, BMI	Age at menarche	Scoliosis, Training load hours: dance, practice	BMI, hyper-mobility	* Relationship of having scoliosis with hyper mobility, BMI, age at menarche and training load<br>* Scoliosis in 9 dancers, scoliosis in 1 non-dancer - sign difference in presence of scoliosis between two groups (p=.006)<br>* Homogeneity between both groups for physical measures except hyper mobility-Brighton's score> 4 in 21 dancers cw1 non dancer (p=.04)<br>* Scoliosis and training load, trend towards a sign difference; dance practice and presence of scoliosis increased with higher hours unsupervised.
**Kim and Lim [93] 2014**	Gymnasts<br>Female	Cross-sectional	N= 22<br>11 Premenarche<br>11 Post Menarche	Years: pre-menarche 11.6 ± 2.2 yrs and post menarche 19.14 ± 3.2 yrs	Training history:<br>Pre-menarche: 37.8 ± 24.4 months and Post-menarche: 123.4 ± 62.2 months	CA, weight, height	Pre and post menarche (NS)		ACL injury risk via max knee flexion, knee abduction, max internal tibial rotation angle, max knee abduction moment.	* ACL injury risk factors between the two groups: the post-menarche group showed a decrease in the maximum knee flexion angle (p =.019) and an increase in the maximum knee abduction angle (p =.039), maximum internal tibial rotation angle (p = 0.043), maximum knee abduction moment (p =.049), and H-Q ratio (p =.033) c/w pre-menarche group<br>*The post-menarche group exhibits an increased risk of noncontact anterior cruciate ligament injury due to their greater knee loads than those of the pre-menarche group
**Steinberg et al. [87] 2023**	Dancers<br>Ballet	Cross-sectional	N=132<br>3 groups:<br>39 12 years<br>40 13 years<br>53 14 years	Years:<br>12-14	Dancers:<br>Mean 13.3hrs/week	Ca, weight, height, BMI	Age at menarche	Joint ROM, muscle strength	Training history	*Height and weight at 13years was sign higher cw 12years (p < .001 and p = .003, respectively), and 14 years cw with 12years (p < .001 and p < .001, respectively). BMI at 14years was sign higher cw age 12 (p < .001).<br>*Hours of practice previous year and hours of practice current year at 14 years were sign higher cw to age 12 (p < .001 and p = .015, respectively)<br>* Pre-menarche dancers had higher joint ROM cw with post-menarche dancers; dancers at post-menarche were stronger compared to dancers at pre-menarche. Slope coefficient was negative at 12yeas in hip ER and en-pointe (−0.80 and −0.52, respectively) and became steeper with age (age 13: −3.52 and −3.28, respectively; age 14: −6.31 and −4.42, respectively).
**Saejong et al. [88] 2021**	Gymnastics-Rhythmic	Cross-sectional	N=28<br>10 gym national<br>18 backup team	Years:<br>16.1±3.0	Training history:<br>8.1 ±3 years<br>Hrs/week NR	CA, weight, height, BF%, LBM	Indirectly via body composition	Scoliosis<br>LBP,<br>Pelvic tilt, hip and Lower leg ROM	Training history, Lumbar muscle strength	* Participants with scoliosis (n=17) sign higher in age (17.0 ±3.0 versus 14.6 ±2.3 years, p = 0.036), height (155.7 ±5.1 vs 153.5 ±5.9 cm, p = 0.009), weight (44.3 ±5.4 vs 38.7 ±7.1 kg, p = 0.001) and BF% 18.1% ±1.7% vs 13.9% ±3.1%, p = 0.000)<br>* Six of the scoliosis group gymnasts had lumbar compression fractures or spondylolysis and back pain VAS (4.29 ±1.90 vs 0.45 ±1.04, p = 0.000)<br>*Lateral flexors Lx showed noticeable imbalance and was more severe in scoliosis<br>*Elite rhythmic gymnasts increasingly susceptible to scoliosis and other spinal disorders with age and years of training

Alison S Fitch, Jocelyn Mara, Felicity Lord, Gordon Waddington. Corresponding author: Alison Fitch Email: sportsphysio@alifitch.com ali.fitch@canberra.edu.au Research Institute for Sport and Exercise, University of Canberra ACT 2617, Australia

Supplementary file S1. (continued)

Training and Performance										
**Baxter Jones et al. [115] 1994**	Gymnasts Female	Mixed cross-sectional/ observational over 3 years	N= 222<br>81 gym<br>60 swimming<br>81 tennis	Years:<br>5 age cohorts 8, 10, 12, 14 and 16 (pre-pubertal, pubertal and post pubertal)	Gym:<br>13.09hrs/week	CA, 4 skinfold sites measured	Age of menarche, mothers age of menarche	Weekly training hours, socioeconomic class		* Mothers and daughters menarcheal age were positively related (p<.01)<br>* Gymnasts trained sign more hours 13.09hr/week than tennis players, 9.7hr/week, not sign more than swimmers, 12.4h/week<br>* Sport and maternal menarcheal age were only independent variable found to have a sign effect (p<.05)on a child's menarcheal age<br>*Sport, maternal menarcheal age, socio-economic class and hours trained showed gym sign(p<.01) later menarche(mean age: 14.2) than swimmers (13.4) and tennis players (13.4)
**Georgopoulos et al. [105] 2011**	Gymnasts- Artistic Female and Male	Case-control	N= 239	Years:<br>gym<br>16± 1.6 (females)<br>16.8 +1.1yrs (males); Pubertal adolescents<br>16 ± 1.4yrs (females)<br>15.3 ± 2yrs (males)	European Championship gym: training suggested ~30hrs/week	CA, weight, height, BF%, LBM	Bone age, Tanner scale, Age at menarche	Salivary samples, Salivary cortisol concentrations measured by electrochemiluminescence quantitation.	Stress questionnaire- specific for study, 4 items rated on an 11 point numerical scale (0-10).	*Female gym higher salivary AM (p<.05) and reported higher degree psychological stress (p<.013)than female aged, matched controls<br>*Male gym had higher reported psychological stress to controls (p<.001) but AM salivary levels did not differ<br>*Female gym had sign higher cortisol levels in AM and PM (p<.01,p<.01 respectively)and higher degree of psychological stress(p<.003) than male gym<br>*Main factors influencing AM cortisol were menarche (p<.05) and BA (p<.05) and PM CA (p<.01)<br>* The female AG were shorter and lighter than average, with mean ht and wt SD scores below 0, delay in BA (p<.001)<br>*Male gym also had lower BF% (8.4% versus 14%) and higher LBM (55% versus 36%) than female (p<.001)
**Lindholm et al. [106] 1994**	Gymnasts Female	Longitudinal observational for 5 years	N=50<br>28 gym<br>22 controls	Years:<br>11-14	Gymnasts: Training since age 7-8years, 10-20hrs/week. training besides school activities.	CA, weight, height, BMI, BF%	Tanner scale, Age at menarche	Physical injuries Menstrual rhythm (including FSH, LH, TSH and prolactin in serum)	Amount of training	* Two gym with slowest pubertal development also reached highest levels in national and international competitions having the latest menarche.<br>*Growth is slower in gym; growth spurt usually occurs in periods of reduced training and often connected to injuries<br>* Gym were shorter and lighter with significant less BF than control group (6.5± 2kg; 7.7 ± 1.7kg); gym had sign delayed menarche (14.5± 1.4); only 7 gymnasts had regular periods<br>* Hormone analysis was consistent with those not yet menstruating<br>* Only 5 gym escaped nil injuries throughout the years, either dropped out or change sports, 6 continued with elite despite injuries<br>* LBP most common complaint, 2 girls developed an eating disorder
**Theintzet al. [107] 1993**	Gymnasts Female	Prospective cohort	N=43<br>22 gym<br>21 controls	Years:<br>Gym 12.3<br>Controls 12.3	Gym:<br>18 - 26 hr/week Swimmers: 4-15hrs/week(av 8 hrs).	CA, weight, height, sitting height, BF%		Training during puberty		*Gym on average were shorter and thinner; sign delay in mean BA (p=.01)<br>*Gym (>18hs/wk) starting before puberty and maintained can alter growth rate so that full adult height won't be reached<br>* Menarcheal age for gym (14.4± 1.2yrs,n=11), swim12.9± 0.9yrs, n=15)<br>* Predicted height as a function of BA, gym decreased sign (same with measured hts, pubertal stages)<br>* Stunting of mean leg length in gym at 12years BA c/w swimmers<br>* Mean growth velocity expressed as a function of BA sign lower in gym than in swimmers from 11-13years; PHV was 8 ± 0.5 cm/yr at a mean bone age of 12yrs in swimmers, in gym PHV was 5.48 ± 0.32cm/yr at a mean BA of 12.5years

Alison S Fitch, Jocelyn Mara, Felicity Lord, Gordon Waddington. Corresponding author: Alison Fitch Email: sportsphysio@alifitch.com ali.fitch@canberra.edu.au Research Institute for Sport and Exercise, University of Canberra ACT 2617, Australia

Supplementary file S1. (continued)

Daly et al. [108] 1998	Gymnasts Male	Case-control study, conducted over 10 month period	N=33 16 gym 17 controls	Mean Years: 10.5	Victorian institute of Gym: >10 h/week (mean 17.2 ± 5.6)	CA, weight, height	Tanner scale, serum total testosterone	Growth: IGF-1:C and T:C ratios	Nutrition intake, training volume/ intensity with HR	* Adjusting for CA, gym were shorter(P<.01) and lighter (P<.05) than control group, no difference in rate of growth or body mass over course of study, gym remained sign shorter throughout<br>* Gym training: 1 min 43 sec rest to 1 min work time, training most demanding during S &C phase then routine development(RD) phase and then pre- comp (PC) phase<br>*Cortisol levels were significantly higher in the gym during the S&C phase of training when c/w RD or PC training<br>* T:C ratio decreased sign from PC to SC training in gym<br>*The average protein intake of the gym was sign greater than controls ($p < 0.05$)
Moeskops et al. [109] 2020	Gymnasts-Artistic Female	Cross-sectional	N= 120	Years: 5-14	Gym: > 1 yr gym experience and participating in gym training 2-6 x week (2-24hrs /week).	CA, Standing weight, height, sitting height	Biological maturity: %PAH	Strength measures / take off velocity	Training history	* Absolute peak force (and force at various time epochs) were sign greater in more mature gym , although no sign differences were observed in relative peak force<br>* Sign differences between competitive levels for absolute rate of force development, absolute peak rate of force development, relative peak rate of force development, take-off velocity<br>* Biological maturation seems to impact isometric force-time curve characteristics in young female gym, and higher-level gym produce greater RFD than those competing at a lower level
Purenović-Ivanović et al. [110] 2017	Gymnasts-Rhythmic Female	Prospective cohort study	N= 126 Five age categories:22 beginners, 38 intermediate, 26 advanced, 25 juniors, 15 seniors	Years: beginners, 7–9 years; intermediate, 9–12 years; advanced, 12–14 years; juniors, 14–16 years; seniors, 16 years and older)	Gym: national and/or international level, training experience 6 months-14years, and 10-16hrs/ week	CA, weight, height, BMI	Tanner scale, Age at menarche	Performance		* Sign influence of gym pubertal development on success considering the sample in total ($p < 0.001$)<br>*Pubertal development sign influenced performance but only explained 14% variance<br>* Axillary hair development had a positive influence on gym performance scores ($p = .03$)<br>* In gym, the established negative relations between the three examined pubertal development parameters and the performance scores could indicate that late maturation is desirable in gym
Richards et al. [111] 1999	Gymnasts- Female	Mixed longitudinal (observational) over 3.3 years	N=37 15 gym high training 30 gym mod training	Years: 10-13.5	Gym high volume: > 20 hrs(mean 30), Gym moderate volume: < 20 (mean15 hours)	CA, weight, height, R body segment lengths, skinfolds equations to calculate growth rate @ 1 month intervals	Age at menarche	Strength variables	Performance tests	*High volume gym were shorter, lighter and smaller than moderate volume gym<br>*Absolute strength levels rose with maturity, as did normalised strength scores with increased age<br>* High volume gym had greater average rotational velocity due to smaller amounts of angular momentum at take-off; angular momentum produced on take-off for maximal front rotation increased with age<br>* high volume gym had greater mean trunk angular velocities during backward rotation, higher angle of take-off, higher centre of mass during flight angular momentum; take off angle and average height of centre of mass achieved during backward rotation increased with age<br>* high volume group had higher amount of rotation around longitudinal axis across each age group, faster v-sit

Supplementary file S1. (continued)

**Tringali et al. [112] 2014**	Gymnasts- Artistic<br>Female	Case-control	N= 58<br>16 elite<br>16 recreational<br>18 synchronised swim	Years: 9-15	16 elite gym:<br>18 ± 4 hrs/week	CA, weight, height, BMI BF%, %muscle	Age at menarche	Training/week, Years of practice	Hormone status; Cytokine profile/immune response	* Increase of IL-6 and TNF- mRNAs in elite female artistic gym due to increased intensity and duration of training than recreational level artistic gym (+73% and +39% respectively; p< .05)<br>* The average levels of circulating 17-β-estradiol were 1.93-fold higher in athletes who reached menarche (age:14 ± 1.6) in comparison to pre-pubertal athletes (age:11 ± 1)<br>* In gym and swim, IL-6 expression appeared to be modulated by the levels of circulating oestrogen: prepubertal athletes revealed a higher increase in IL-6 than pubertal<br>* In pre-pubertal athletes, BMI percentile was inversely correlated with the increase of IL-6 and TNF-α mRNAs expression in PBMCs (p<.05)<br>Conclusion: These changes shifted cytokine profile towards pro-inflammatory status- may negatively affect growth of female athlete
**Castelo-Branco et al. [113] 2006**	Dancers-<br>Ballet<br>Female	Case-control	N= 115<br>38 Dancers<br>77 high school girls	Years:<br>Dancers<br>14.8±±± 1.7<br>High school girls<br>14.8 ± 1.6	Dancers:<br>>1year at dance school<br>15.5 hrs/week	CA, weight, height	Age at menarche	Training level/intensity	Nutrition intake	* Average age of menarche was 12.4yrs(12-12.8)dancers, 12yrs(11.8-12.3) controls<br>*Dancers higher prevalence of oligomenorrhoea (34% vs 14%) and amenorrhea (8% vs 11%)than controls<br>* % weight was sign lower in dancers (18% underweight vs 2.6% controls)<br>*BMI: 21% in dancers vs 13% controls<br>* 32% dancers weight control diet vs 12% controls, 76% of dancers did not take four daily meals where in the control was 56%.
**Pourmotahari et al. [103] 2022**	Gymnasts-<br>Artistic<br>Female	Cross-sectional	N= 60<br>30 gym<br>30 untrained	Years: 9-18<br>3 groups:<br>Tanner 1; Tanner 11; Tanner 111 and adult)	NR	CA, weight, height, BF%, skin folds	Tanner scale	Strength measures, Neuromuscular changes (RMS from EMG)	Training	* Gym was stronger than untrained when absolute torque adjusted for weight (p<0.05)<br>* The interaction of age group and training history was not sign different in all variables but main effect of training history on RMS was sign (p<0.001)<br>*RMS (p<0.001), normalized RFD (P<0.05) and vertical jump (P<0.05) of gym groups were sign > than UT, but no sign difference b/n age groups for RMS and normalized RFD<br>* Regular gymnastics training during pre-puberty period seems to preserve this pattern of changes at a higher level compared to untrained girls.
**Gómez-Dolader et al. [104] 2024**	Gymnasts-<br>Acrobatic<br>Female	Cross-sectional	N= 34	Years:<br>Circa PHV<br>11.92±1.67<br>Post PHV<br>16.47±1.80	Gym:<br>13.5hrs/week	CA, weight, height, BMI	PHV/maturity offset	Strength measures, Balance tests	Training history	* Post-PHV group performed better in most strength and balance measures (except CMJ height, p = 0.083, and TTS, p = 0.926)<br>* Height and body mass negatively correlated with CoP excursion, height showed positive correlations with strength in circa-PHV but not post-PHV (p = 0.014 to 0.057)<br>* Maximal isometric strength and CMJ power were strongest predictors of static standing balance, with stronger predictive strength in circa-PHV group<br>*Strength-balance relationships varied by maturation and balance task type (static-proactive vs. standing-inverted).

Abbreviations: gym (gymnast); chronological age (CA); height (ht); weight(wt); body mass index (BMI); body fat percentage (BF%); fat free mass (FFM); fat mass(FM); lean body mass (LBM); bone age (BA); predicted adult height (PAH); Calcium (Ca); magnetic resonance imaging (MRI); physical exam (PE); blood pressure (BP); peripheral quantitative computed tomography (pQCT); body weight (BW); counter movement jump(CMJ); plantar flexor's (PF's); bone mineral density (BMD); areal bone mineral density (aBMD); volumetric BMD (VBMD); bone mineral content (BMC); bone strain index (BSI); compressive strength index(CSI); dual-energy X-ray absorptiometry(DXA); speed of sound (SOS); dominant (dom); non dominant (non dom); broadband ultrasonic attenuation (BUA); cortical thickness (CRTHK); polar stress strength index in torsion (SSIp); plasma intact (iPTH); rate of force development (RFD); weight bearing (WtB); lumbar spine (Lx); whole body (WB); total body (TB); femoral neck (FN); radius (R); wards triangle (WT); ultra distal radius (UDR); low back pain (LBP); skinfolds (SF); compared with (c/w); significant (sign); of regression (OR); gynage (gynaecological age); Australian ballet school (ABS); Ultrasound tissue characterisation (UTC); recommended daily allowance (RDA); peripheral blood mononuclear cells (PBMCs); interleukins (IL-6, IL-1); tumour necrosis factor alpha(TNF-α); messenger ribonucleic acid (mRNA); insulin-like growth factor 1(IGF-1); testosterone: cortisol (T:C); dehydroepiandrosterone sulphate (DHEAS); low energy availability (LEA); LEA in females questionnaire (LEAF-Q); electromyography (EMG); Eating disorder (ED); brief eating disorder in athletes questionnaire (BEDA- Q) General joint hypermobility (GJH); root mean square(RMS); time to stabilization(tts); rate of force development(RFD)
