# Supplementary material for: Key Factors Impacting Performance Health During Growth and Maturation in Adolescent Competitive Aesthetic and Acrobatic Athletes: A Systematic Review
Source: Sports Med. 2026 Mar 27;56(7):1701–21. doi: 10.1007/s40279-026-02416-5 (PMC13388506; doi:10.1007/s40279-026-02416-5)
Supplement: Supplementary file 2 — Supplementary file2 (PDF 100 KB) [file 40279_2026_2416_MOESM2_ESM.pdf]

Key factors impacting performance health during growth and maturation in adolescent competitive aesthetic and acrobatic athletes: A systematic review. Sports Medicine.
Alison S Fitch, Jocelyn Mara, Felicity Lord, Gordon Waddington. Corresponding author: Alison Fitch Email: sportsphysio@alifitch.com ali.fitch@canberra.edu.au

Research Institute for Sport and Exercise, University of Canberra ACT 2617, Australia

Supplementary file 2: Search string for each database

Web of Science Core Collection

TS = (diving OR dive* OR FINA near/5 "aquatic sports" OR sport* OR "olympic near/2 sport*" OR aesthetic sport* OR acrobatic sport* OR artistic sport* OR gymnastics OR "extreme near/5 sports" OR cheerleading OR cheer OR aerial near/5 ski* OR "snow near/5 sports" OR surf* OR "ice near/3 skat*" OR dance OR danc*)NOT TI=(scuba OR deep-sea OR decompression OR hypobaric)NOT AB=(scuba OR deep-sea OR decompression OR hypobaric) TS = (adolescent OR young adult* OR teen* OR youth* OR athlet*)TS= (injur* OR illness OR ill OR sick* OR overuse injur* OR acute injur* OR "health near/2 problems" OR pain OR stress OR trauma OR mental OR psych OR predictor OR risk OR perform*)TS = (grow* OR maturat* OR "peak height velocity" OR height OR weight OR pubert* OR skeletal near/2 age OR "wrist near/2 X-ray")NOT TS = (obesity OR diabetes or cardiovascular) NOT TS= (soccer OR football OR handball OR basketball)NOT TS= (recreation OR leisure)

Pub Med

(((diving OR dive* OR FINA OR "aquatic sports" OR sport* OR "olympic sport*" OR aesthetic sport* OR acrobatic sport* OR artistic sport* OR gymnastics OR "extreme sports" OR cheerleading OR cheer OR aerial ski* OR "snow sports" OR surf* OR "ice skat*" OR dance OR danc* NOT (scuba OR deep-sea OR decompression OR Hypobaric)) AND ((adolescent* OR young adult* OR teen* OR youth* OR athlet*))) AND ((injur* OR illness OR ill OR sick OR overuse injur* OR acute injur* OR "health problems" OR pain OR stress OR trauma OR mental OR psych OR predictor OR risk OR perform*))) AND ((grow* OR maturat* OR "peak height velocity" OR height OR weight OR pubert* OR skeletal age OR "wrist X-ray" OR X-ray*)) AND AND (adolescent[Filter] OR child[Filter]) AND NOT (obesity OR "school child*" OR recreation*) AND NOT (soccer OR football OR run* OR basketball OR handball) AND NOT (cardiovascular OR diabetes OR leisure OR asthma)

Alison S Fitch, Jocelyn Mara, Felicity Lord, Gordon Waddington. Corresponding author: Alison Fitch Email: sportsphysio@alifitch.com ali.fitch@canberra.edu.au

Research Institute for Sport and Exercise, University of Canberra ACT 2617, Australia

Supplementary file 2: Search string for each database (continued)

SPORTdiscus

(diving OR dive* OR FINA adj5 Aquatic sport* OR sport* OR olympic adj2 sport* OR aesthetic sport* OR artistic sport* OR acrobatic* OR gymnastic* OR extreme adj2 sport* OR cheerleading OR cheer OR aerial adj5 ski* OR snow adj5 sport* OR surf* OR ice adj2 skat* OR danc*) NOT (scuba OR deep-sea OR decompression OR Hypobaric) (adolescent* OR youth* OR young adj2 adult OR athlet* OR athlete*) (injur* OR illness OR ill OR sick* OR overuse injur* OR acute injur* OR "health adj/2 problems" OR pain OR stress OR trauma OR mental OR psych OR predictor OR risk OR perform*) grow* OR maturat* OR peak height velocity OR height* OR weight* OR pubert* OR skeletal adj2 age OR wrist adj5 X-ray) NOT (obesity OR childbirth OR diabetes OR cardiovascular) NOT (soccer OR football OR handball OR basketball) NOT (recreation OR leisure OR school adj2 children)

PsycINFO

(diving OR dive* OR FINA adj5 Aquatic sport* OR sport* OR olympic adj2 sport* OR aesthetic sport* OR artistic sport* OR acrobatic* OR gymnastic* OR extreme adj2 sport* OR cheerleading OR cheer OR aerial adj5 ski* OR snow adj5 sport* OR surf* OR ice adj2 skat* OR danc*) NOT (scuba OR deep-sea OR decompression OR Hypobaric) (adolescent* OR youth* OR young adj2 adult OR athlet* OR athlete*) (injur* OR illness OR ill OR sick* OR overuse injur* OR acute injur* OR "health adj/2 problems" OR pain OR stress OR trauma OR mental OR psych OR predictor OR risk OR perform*) grow* OR maturat* OR peak height velocity OR height* OR weight* OR pubert* OR skeletal adj2 age OR wrist adj5 X-ray) NOT (obesity OR childbirth OR diabetes OR cardiovascular) NOT (soccer OR football OR handball OR basketball) NOT (recreation OR leisure)
